# Overexpressed MET drives aggressive thyroid cancer phenotypes and serves as a precision therapeutic target

**DOI:** 10.1038/s41598-025-23587-7

**Published:** 2025-11-13

**Authors:** Ruyue Xu, Yiqiu Wan, Biran Ding

**Affiliations:** 1 Department of Biochemistry and Molecular Biology, Anhui Medical University, Hefei, 230032 China; 2 The Clinical Laboratory, the First Affiliated Hospital of Anhui Medical University, Hefei, 230011 Anhui China

**Keywords:** MET, Thyroid cancer, Migration, EMT, Cancer, Biomarkers

## Abstract

The global incidence of thyroid carcinoma (THCA) is increasing. Although generally indolent with a favorable prognosis, a subset of cases exhibits aggressive progression. Conventional chemotherapy frequently induces severe systemic toxicity owing to poor target specificity, highlighting the need for more targeted therapeutic approaches. The HGF/c-MET signaling pathway plays a pivotal role in tumorigenesis and disease progression, promoting malignant tumor development through diverse mechanisms, including cell proliferation and migration. By combining integrated multi-omics bioinformatics analysis with functional experiments, this study demonstrates that the *MET* gene represents a promising theragnostic marker for THCA. Specifically, our study demonstrated: (1) The *MET* gene is significantly overexpressed in THCA tissues compared to normal controls; (2) This dysregulation is closely associated with aggressive malignant phenotypes, including enhanced tumor progression, increased metastatic potential, reduced survival, and immune-suppressive characteristics; (3) Retrospective clinical case analysis indicated that the lymph node metastasis rate was significantly elevated in the *MET* high-expression group relative to the low-expression group; (4) RNA interference (RNAi)-mediated *MET* knockdown resulted in a marked decrease in the migratory and invasive capacities of thyroid cancer cells in vitro. Collectively, these findings not only identify MET as a robust molecular classifier for THCA but, more critically, uncover its therapeutic potential as an actionable target within the HGF/c-MET axis for refractory THCA, providing both experimental evidence and a theoretical framework for developing precision diagnostic and therapeutic strategies.

**Supplementary Information:**

The online version contains supplementary material available at 10.1038/s41598-025-23587-7.

## Introduction

Thyroid carcinoma (THCA), which arises from thyroid follicular epithelial cells, represents the predominant endocrine malignancy globally^1–3^, and its incidence has increased steadily in recent decades^1^. Over 95% of THCA cases originate from follicular cells and typically represent indolent tumors amenable to cure through combined surgical resection and radioactive iodine ablation^4,5^. The 5-year survival rates are 93% and 88% for well-differentiated thyroid cancer in female and male patients, respectively^6^. Nevertheless, clinical challenges persist, particularly since a subset of patients is diagnosed with metastatic disease upon initial presentation. Although targeted therapies and immunotherapy are effective in some advanced cases^7,8^, many patients still lack effective treatment options^9^. This unmet clinical need underscores the urgent need for developing innovative therapeutic approaches to improve outcomes in thyroid cancer management.

The hepatocyte growth factor (HGF)/cellular mesenchymal-epithelial transition factor (c-MET) axis plays a pivotal role in diverse physiological processes, such as embryonic development, angiogenesis, and wound healing^10^. In cancer, this signaling cascade may become dysregulated through multiple mechanisms, including c-MET overexpression or gain-of-function mutations in its kinase domain. These aberrations result in constitutive activation of downstream pathways, such as mitogen-activated protein kinase/extracellular signal-regulated kinase (MAPK/ERK), phosphoinositide 3-kinase/protein kinase B (PI3K/AKT), signal transducer and activator of transcription (STAT), and inhibitor of kappa B alpha/nuclear factor kappa-light-chain-enhancer of activated B cells (IκBα/NF-κB). Ultimately, these dysregulated pathways collectively drive tumor proliferation, invasion, migration, survival, and metastasis^11–14^. Notably, both THCA cell lines and clinical specimens display significant c-MET overexpression. Although c-MET is largely absent in normal thyroid tissues, it is markedly upregulated in approximately 70–90% of papillary THCA samples^15–17^. These findings establish c-MET as a highly promising therapeutic target in THCA.

In summary, our pan-cancer analysis of TCGA data identified MET as exhibiting the highest expression level in THCA among all cancer types. Notably, elevated MET expression drives metastatic progression in thyroid cancer, a finding corroborated by clinical specimen analysis revealing a significant correlation between high MET expression and metastatic potential (*P* = 0.0002). Genetic silencing of MET markedly attenuated cancer cell migration capacity, concomitant with downregulation of epithelial–mesenchymal transition (EMT)-related signaling molecules and diminished activation of both STAT3 and ERK pathways. Moreover, we uncovered significant associations between MET expression and immune cell infiltration within the tumor microenvironment. Collectively, these multidimensional findings establish MET as a pivotal regulator of THCA progression, orchestrating metastasis, modulating oncogenic signaling pathways, and shaping the immune landscape.

## Results

### *MET* is upregulated in THCA

**Fig. 1:**
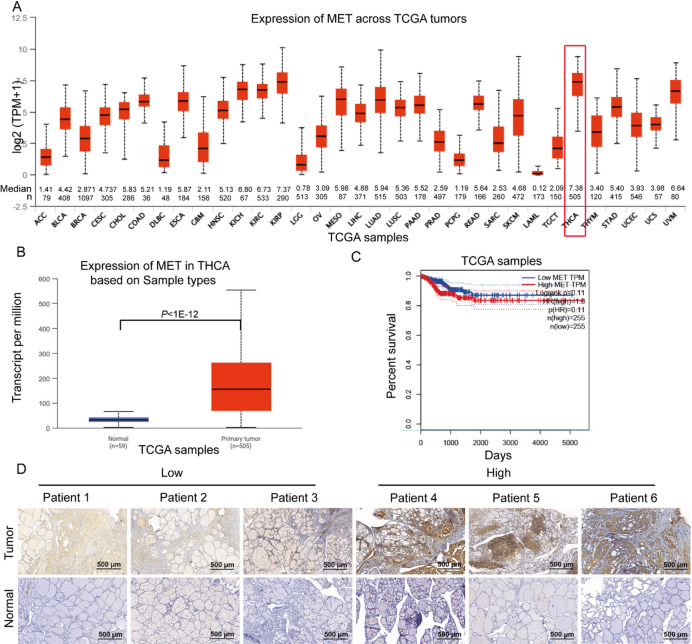
The expression profile of *MET* in THCA tissues. (**A**) Expression profile of MET in different cancers: a UALCAN database analysis. (**B**) TCGA analysis: MET expression in THCA and normal tissue. (**C**) Survival prognosis of THCA patients based on MET expression: an analysis of CEPIA database. (**D**) IHC analysis for the differential expression of MET in THCA tissues, and normal tissues. Patients 1–3 were classified as having low MET expression, while patients 4–6 were classified as having high MET expression. Scale bars = 500 μm.

Utilizing the UALCAN database, we assessed MET expression across multiple cancer types. THCA exhibited the highest MET expression levels (Median = 7.38, *n* = 505) among all evaluated tumors (Fig. 1A). Notably, MET expression was significantly higher in THCA samples (*n* = 505) than in normal thyroid tissues (*n* = 59) (*P* < 1 × 10^−12^, Fig. 1B). Although Kaplan-Meier survival analysis indicated a non-significant trend (*P* = 0.11) toward worse prognosis in patients with high MET expression (*n* = 255) compared to the low-expression group (*n* = 255) (Fig. 1C), immunohistochemical analysis of our independent cohort (31 matched THCA-normal tissue pairs) confirmed consistent MET upregulation in tumors (Fig. 1D). These findings suggest that MET overexpression may contribute to THCA progression.

### Elevated MET expression correlates with metastatic progression in thyroid cancer

**Fig. 2:**
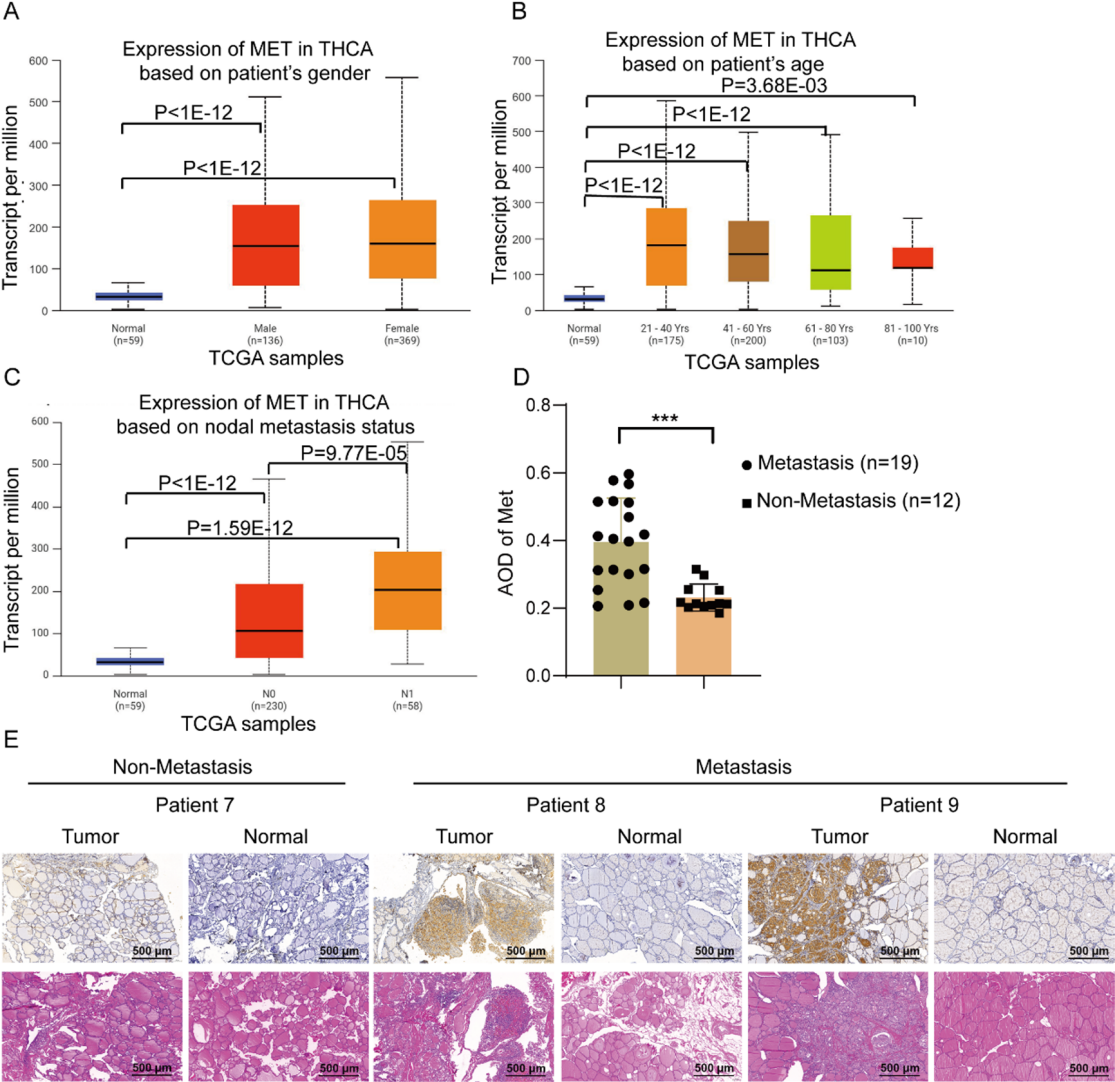
Elevated MET expression correlates with metastatic progression in thyroid cancer. (**A**) Association between MET expression and sex in THCA. (**B**) Association between MET expression and age in THCA. (**C**) Association between MET expression and lymph node metastasis status in THCA. (**D**) Quantitative analysis of MET expression (the average optical density, AOD) using IHC staining, (**E**) IHC analysis of MET expression in THCA tissues, Scale bars = 500 μm. MET expression correlated with metastasis: Patient 7 showed low expression of MET and no metastasis, while Patient 8 and 9 had medium and high expression of MET, respectively, and both exhibited metastases. ***P < 0.001.

Our findings revealed a positive correlation between MET expression and metastatic potential in THCA. Analysis demonstrated significantly higher MET expression in both male (*n* = 136) and female (*n* = 369) THCA patients compared to normal controls (*n* = 59), with no significant gender-based differences among THCA cases (Fig. 2A). Age-stratified evaluation showed consistent MET upregulation across all age groups (21–40 years: *n* = 175, *P* < 1 × 10^−12^; 41–60 years: *n* = 200, *P* < 1 × 10^−12^; 61–80 years: *n* = 103, *P* < 1 × 10^−12^; 81–100 years: *n* = 10, *P* = 3.68 × 10^−2^) compared to normal tissues, but revealed no significant age-dependent variation (Fig. 2B). Strikingly, MET expression increased progressively from normal tissues (*n* = 59) to N0 (*n* = 230, *P* < 1 × 10^−12^) and N1 (*n* = 58, *P* = 1.59 × 10^−12^) cases, with significantly elevated levels in N1 compared to N0 patients (*P* = 9.77 × 10^−3^) (Fig. 2C).

Immunohistochemical validation in 31 clinical specimens (12 non-metastatic vs. 19 metastatic cases) confirmed that high MET expression was strongly associated with metastatic status (*P* = 0.0002) (Fig. 2D,E). Representative cases illustrated increasing metastatic potential with higher MET expression (Patient 7 with metastasis vs. Patients 8–9 without metastasis). Taken together, these results strongly suggest that MET overexpression drives THCA progression and metastasis.

### Knockdown of MET suppresses thyroid cancer cell migration in vitro

**Fig. 3:**
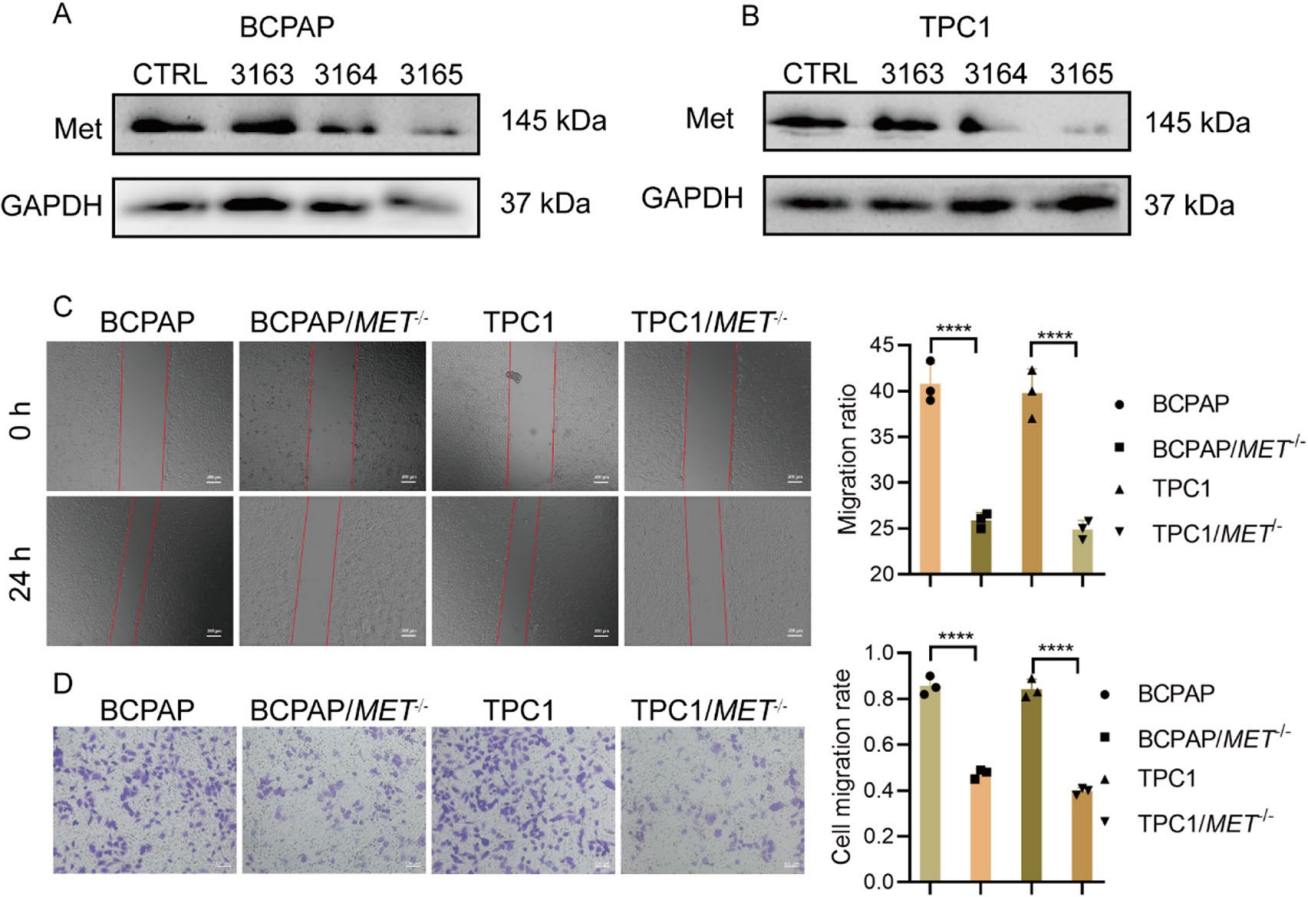
Knockdown of MET inhibits THCA cell migration. (**A**,**B**) Western blotting analysis of MET expression in different cell lines. (**C**) Wound healing assays of cell migration, Scale bars = 200 μm. (**D**) Transwell analysis for cell migration, Scale bars = 100 μm. ****P < 0.0001.

Employing RNAi knockout (sgRNA-3165 demonstrated the highest efficiency, Fig. 3A,B), we established MET-deficient thyroid cancer cell lines (BCPAP/*MET*^−/−^ and TPC1/*MET*^−/−^) for functional analysis. Wound-healing assays demonstrated significantly greater scratch width (24 h) and reduced migration capacity in MET-knockout cells compared to wild-type controls (Fig. 3C). Consistent with these findings, Transwell assays revealed markedly decreased cell migration through membranes (Fig. 3D). Together, these results establish that MET is essential for thyroid cancer cell migration.

### Knockdown of MET inhibits EMT and the ERK and STAT3 signaling pathways

**Fig. 4:**
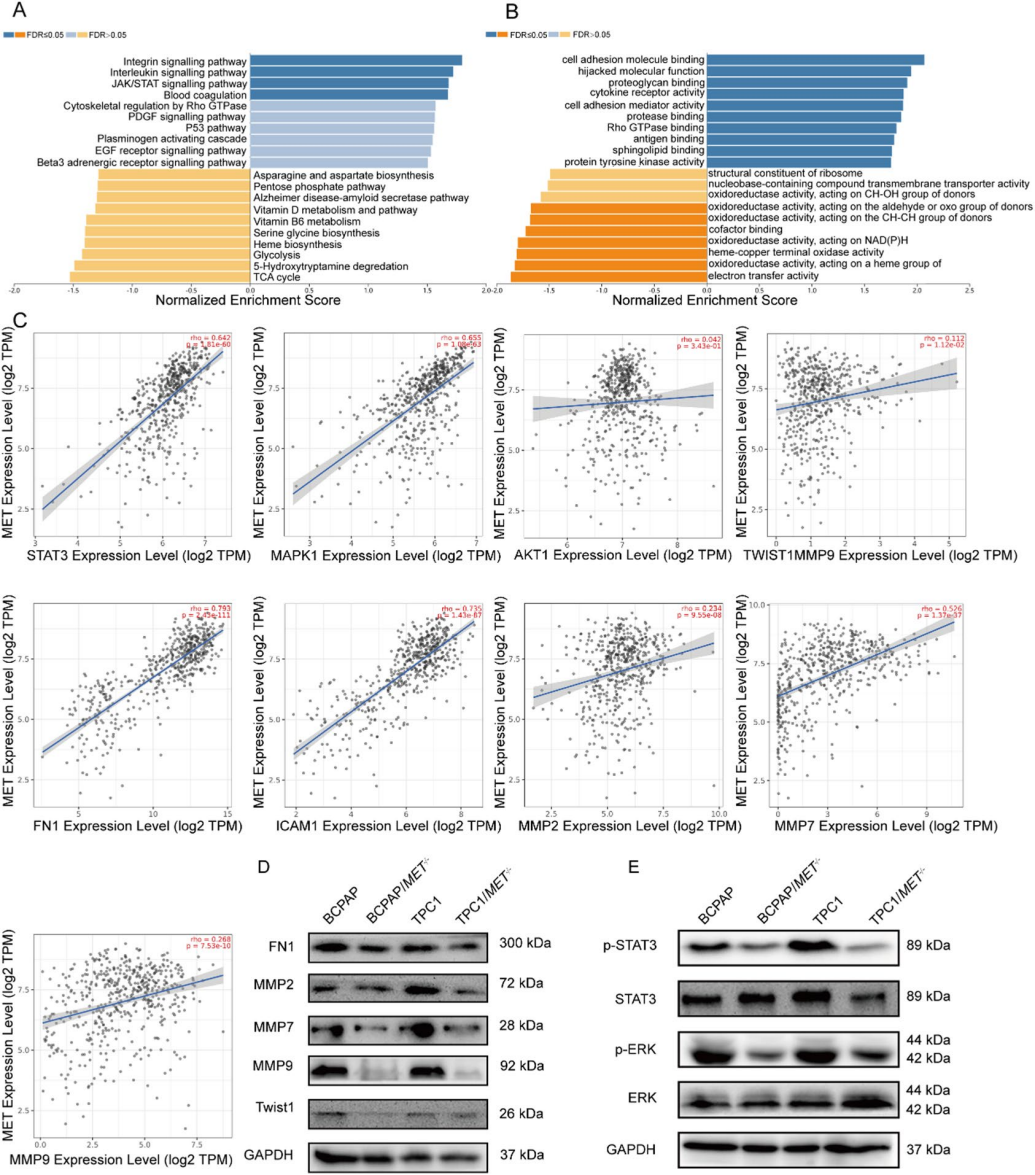
Knockdown of MET suppresses EMT, ERK, and STAT3 signal pathways in THCA cells. (**A**,**B**) Enrichment analysis of the *MET* gene in THCA. (**C**) Correlation analysis between *MET* and *STAT3*, *MAPK1*, *AKT1*, *Twist1*, *FN1*, *ICAM1*, *MMP2*, *MMP7*, and *MMP9* using TIMER2.0. (**D**) Western blot analysis of FN1, MMP2, MMP7, MMP9, and Twist1 in THCA cells. (**E**) Western blot analysis of p-STAT3, STAT3, p-ERK, and ERK in THCA cells.

Gene Set Enrichment Analysis (GSEA) of MET-high THCA using LinkedOmics identified significant enrichment in critical oncogenic pathways, particularly JAK/STAT signaling (NES = 1.72, FDR q < 0.001) and cell adhesion molecule binding (NES = 1.58, FDR q < 0.01) (Fig. 4A,B). Subsequent correlation analysis via TIMER 2.0 revealed strong positive associations between MET expression and key metastasis-related molecules: STAT3 (*r* = 0.642, *P* = 1.81E−60), MAPK1 (*r* = 0.655, *P* = 1.08E−63), AKT1 (*r* = 0.042, *P* = 3.43E−01), Twist1 (*r* = 0.112, *P* = 1.12E−02), FN1 (*r* = 0.793, *P* = 2.43E−111), ICAM1 (*r* = 0.735, *P* = 1.43E−87), MMP2 (*r* = 0.234, *P* = 9.55E−08), MMP7 (*r* = 0.526, *P* = 1.37E−37), MMP9 (*r* = 0.268, *P* = 7.53E−10)(Fig. 4C). In MET-knockout cell lines (BCPAP/*MET*^−/−^ and TPC1/*MET*^−/−^), we observed coordinated downregulation of EMT markers (FN1, MMP2/7/9, and Twist1) at the protein level (Fig. 4D). Mechanistic studies through phosphoprotein profiling demonstrated significant reduction in both STAT3 and ERK pathway activation in MET-deficient cells compared to wild-type controls (Fig. 4E). These findings define a MET-mediated oncogenic axis in THCA progression, wherein MET coordinately activates ERK/STAT3 signaling cascades, enhances expression of EMT-transcription factors, upregulates extracellular matrix remodeling proteins, and collectively driving tumor invasion and metastasis.

### The association between MET expression and thyroid differentiation genes in TCGA

**Fig. 5:**
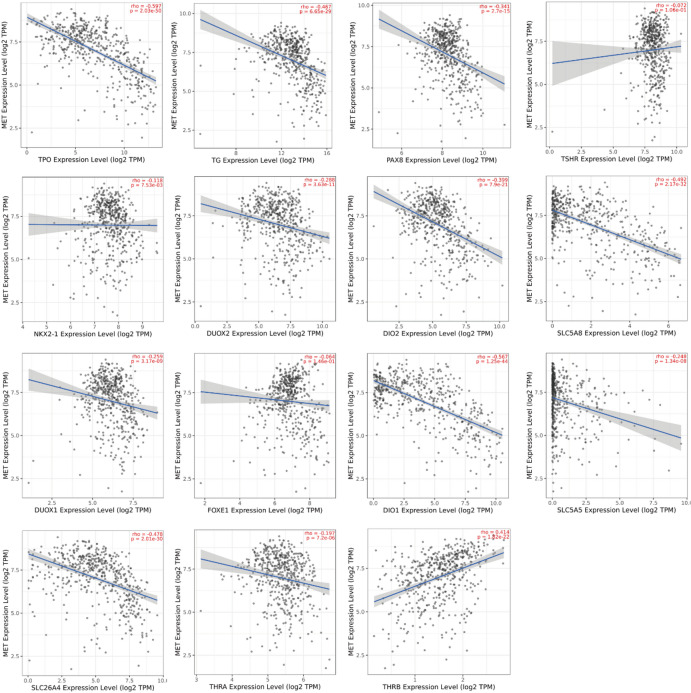
MET is positively correlated with thyroid dedifferentiation degree in THCA. The correlation between MET expression and 15 thyroid differentiation genes was analyzed using the TIMER2.0 database.

Given the critical role of loss of thyroid differentiation in papillary thyroid carcinoma (PTC) progression, we utilized TIMER 2.0 to systematically analyze the association between MET expression and 15 key thyroid differentiation genes. Our analysis revealed significant negative correlations between MET expression and most differentiation markers (Fig. 5), including thyroperoxidase (*TPO*,* r* = − 0.597, *P* = 2.03E−50), thyroglobulin (*TG*,* r* = − 0.467, *P* = 6.65E−29), paired box 8 (*PAX*,* r* = − 0.341, *P* = 2.7E−15), thyrotropin receptor (*TSHR*, *r* = − 0.072, *P* = 1.06E−01), NK2 homeobox 1 (*NKX2-1*,* r* = − 0.118, *P* = 7.53E−03), dual oxidase 2 (*DUOX2*,* r* = − 0.288, *P* = 3.63E−11), iodothyronine deiodinase 2 (*DIO2*,* r* = − 0.399, *P* = 7.9E−21), apical iodide transporter (*SLC5A8*,* r* = − 0.492, *P* = 2.17E−32), dual oxidase 1 (*DUOX1*, *r* = − 0.259, *P* = 3.17E−09), forkhead box E1 (*FOXE1*,* r* = − 0.064, *P* = 1.46E−01), iodothyronine deiodinase 1 (*DIO1*,* r* = − 0.567, *P* = 1.25E−44), sodium iodide symporter (*SLC5A5*,* r* = − 0.2488, *P* = 1.34E−08), pendrin (*SLC26A4*,* r* = − 0.478, *P* = 2.01E−30), and thyroid hormone receptor alpha *(THRA*,* r* = − 0.197, *P* = 7.2E−06). Notably, MET expression showed a unique positive correlation with thyroid hormone receptor beta (*THRB*,* r* = 0.414, *P* = 1.82E−22). These results demonstrate that elevated MET expression is strongly associated with reduced expression of thyroid differentiation markers, suggesting MET may drive tumor dedifferentiation in THCA. Collectively, our findings support a model wherein MET upregulation promotes THCA progression and metastasis by inducing a dedifferentiated state.

### The correlation between *MET* expression and immune cell infiltration in THCA

**Fig. 6:**
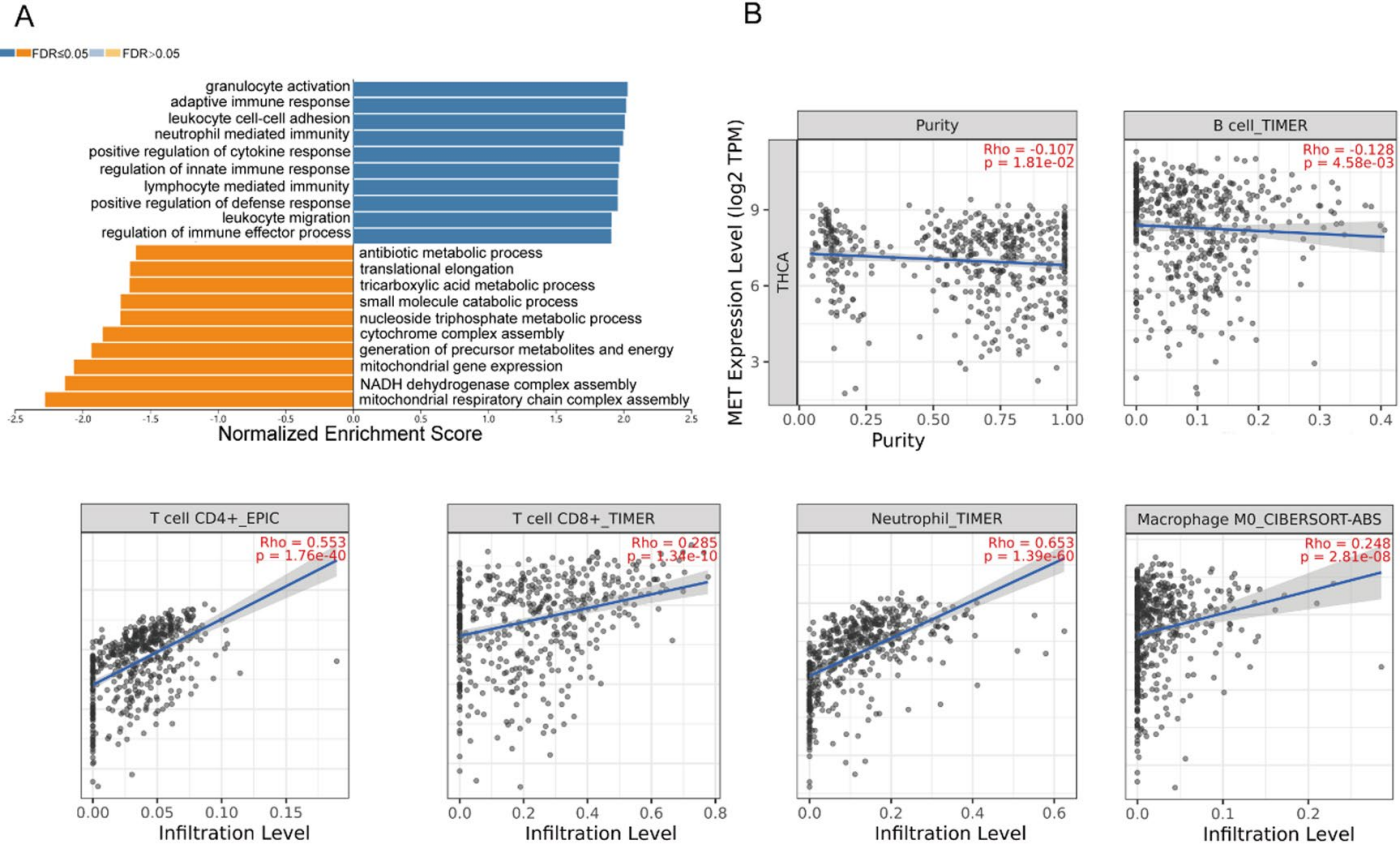
*MET* was correlated with immune infiltration. (**A**) Enrichment analysis of the *MET* gene in THCA. (**B**) The correlation of MET expression with tumor purity and six immune infiltrates (B cells, CD4^+^ T cells, CD8^+^ T cells, neutrophils, and macrophages) was estimated by TIMER in THCA.

GSEA of MET-high-expressing THCA using the LinkedOmics database identified significant enrichment in immune-related processes, most notably regulation of immune effector processes (Fig. 6A). Considering the critical role of the tumor immune microenvironment in cancer progression, we evaluated the correlation between MET expression and immune infiltration levels in THCA via the TIMER database. Remarkably, MET expression exhibited strong positive correlations with the abundance of tumor-infiltrating immune cells, including CD4^+^ T cells, CD8^+^ T cells, neutrophils, and macrophages (all *P* < 0.0001, Fig. 6B). These findings reveal a significant association between MET expression and immune cell infiltration in THCA, suggesting its potential involvement in modulating the tumor immune microenvironment.

## Discussion

Thyroid cancer remains the most common endocrine malignancy, with its incidence having increased steadily in recent decades. Although improved diagnostic techniques may partly explain this trend, a true increase in both incidence and mortality cannot be ruled out^18,19^. Metastatic disease represents the main cause of THCA-associated morbidity and mortality^20^, affecting approximately 10% of papillary and up to 25% of follicular THCA patients^21,22^. The lungs (80%) and bones (25%) are the most frequent metastatic sites, and patients with multiple-organ metastases experience a sharp decline in 5-year survival rates from 77.6% to 15.3% compared to those with single-organ involvement^23^. These findings highlight the urgent need to better understand the molecular mechanisms underlying THCA progression, which may facilitate earlier detection and novel therapeutic approaches for advanced cases.

Numerous studies have demonstrated the oncogenic role of MET overexpression in various malignancies, including: mediating chemoresistance in non-small cell lung cancer^24^, conferring tamoxifen resistance in breast cancer^25^, promoting gastric cancer progression^26^, and facilitating hepatocellular carcinoma initiation and metastasis^27,28^. Our comprehensive pan-cancer analysis identified THCA as exhibiting the highest MET expression levels, with overexpression associated with shorter survival. It should be noted that the survival analysis of MET in the TCGA cohort lacked statistical significance, which may reflect inherent limitations of retrospective datasets, including insufficient follow-up duration, unaccounted treatment heterogeneity, or inadequate statistical power to detect subtle survival differences. Future prospective validation in treatment-standardized cohorts with extended follow-up is warranted to clarify the prognostic value of MET. These collective findings establish MET as a highly promising therapeutic target in THCA, meriting further mechanistic exploration and clinical development.

Metastasis constitutes a major cause of mortality in THCA^23,29^. Our multidisciplinary investigation elucidates MET’s pivotal role in THCA progression through three complementary lines of evidence: (1) Bioinformatic analysis of TCGA data established a significant association between MET overexpression and lymph node metastatic burden; (2) Histopathological validation in clinical specimens confirmed enhanced metastatic potential in MET-high tumors; (3) MET knockout in THCA cells substantially impaired migratory capacity. Complementary GSEA analysis linked MET to cell adhesion, EMT processes, and MAPK/STAT pathway activation. Notably, while the canonical HGF/c-MET pathway activates PI3K/AKT signaling, our study found no significant correlation between MET and AKT1 expression in thyroid cancer, suggesting tissue-specific pathway rewiring. Instead, MET appears to preferentially signal through STAT3 and ERK/MAPK, as evidenced by: (1) elevated phospho-ERK in MET-high tumors (Fig. 4E); (2) strong MET-STAT3 target gene correlations^30^ (Fig. 4C,E); and (3) epigenetic silencing of PIK3R1 that may decouple MET from PI3K signaling^31^. The maintained AKT homeostasis could be attributed to PTEN-mediated feedback or compensation by other AKT isoforms^32^. Importantly, measuring total AKT1 mRNA levels rather than phosphorylation status may underestimate pathway activity. These findings highlight the context-dependent nature of MET signaling and its alternative pathway utilization in thyroid cancer progression. Furthermore, MET expression showed coordinated regulation with established metastatic mediators (FN1, Twist1, ICAM1, MMP2/7/9), and its genetic suppression not only downregulated these effectors but also inhibited MAPK/STAT3 signaling. These integrated findings collectively establish MET as a key regulator of THCA metastasis, operating through MAPK/STAT3-mediated orchestration of metastatic effector molecules. This work provides a compelling mechanistic rationale for targeting MET in advanced THCA.

Loss of thyroid differentiation markers and impaired iodine avidity represent hallmark features of radioiodine-refractory THCA^33,34^. Analysis of TCGA data revealed significant co-expression patterns between MET and 14 key thyroid functional genes (including *PAX8*, *TG*, *TPO*, *TSHR*, *NKX2-1*, *SLC5A5*, *SLC5A8*, *SLC26A4*, *FOXE1*, *THRA*, *DUOX1*, *DUOX2*, *DIO1*, *DIO2*), while demonstrating a negative correlation with *THRB*. These genes are involved in iodine metabolism and thyroid-specific transcription, implicating MET in the regulation of thyroid cancer differentiation. The upregulation of *THRB* may act as a compensatory mechanism against MET-driven dedifferentiation, potentially mediated by MAPK-induced transcriptional activation of *THRB* or feedback resulting from reduced thyroid hormone production. Accumulating evidence underscores the prognostic significance of the tumor immune microenvironment in multiple cancers^34^. TIMER database analysis demonstrated significant positive correlations between MET expression and immune cell infiltration, particularly of CD8^+^ T cells, macrophages, and neutrophils in THCA.

## Materials and methods

### Data acquisition

Pan-cancer analysis of MET expression was performed using the TCGA database (https://cancergenome.nih.gov/). Differential expression of MET between normal and THCA tissues was assessed via the UALCAN database (https://ualcan.path.uab.edu/). The correlation between MET expression and THCA prognosis was evaluated using the GEPIA2 database (http://gepia2.cancer-pku.cn/#index). GSEA was conducted using the Hallmark and Kyoto Encyclopedia of Genes and Genomes (KEGG) gene sets (available at http://www.genome.jp/kegg/)^35–37^. Statistical significance was determined using the Benjamini–Hochberg method to adjust P-values, with thresholds set at an adjusted *P* < 0.05 and a false discovery rate (FDR) < 0.05.

### Analysis of the thyroid differentiation genes

To explore potential functional links, we examined the relationship between MET expression and 15 key thyroid differentiation genes (*PAX8*,* TG*,* TPO*,* TSHR*,* NKX2-1*,* SLC5A5*,* SLC5A8*,* SLC26A4*,* FOXE1*,* THRA*,* THRB*,* DUOX1*,* DUOX2*,* DIO1*,* and DIO2*) using Spearman’s rank correlation analysis via Timer2.0 (http://timer.cistrome.org/). All correlation analyses were corrected for multiple testing using the Benjamini–Hochberg false discovery rate (FDR) method.

### Human tissue samples

Paired THCA specimens (*n* = 31, including 19 metastatic and 12 non-metastatic cases) along with their matched normal tissues (sampled ≥ 2.5 cm from tumor margins) were collected from treatment-naïve THCA patients during surgical resection at The First Affiliated Hospital of Anhui Medical University. This study was approved by the Ethics Committee of the First Affiliated Hospital of Anhui Medical University (Approval No. 20231337) and was conducted in compliance with the Declaration of Helsinki. All participants provided written informed consent.

### Immunohistochemistry (IHC)

Formalin-fixed, paraffin-embedded (FFPE) tissue section (5 μm) were adhered to anti-detachment slides, dewaxed with xylene, and rehydrated through an ethanol gradient. Antigen retrieval was performed by heating the sections in citrate buffer (15 min, 95–100 °C) followed by natural cooling. Endogenous peroxidase was inactivated with 3% H_2_O_2_ (20 min, PV-9000 kit, reagent 1). Following serum blocking (20 min), slides were incubated with a primary anti-MET antibody (1:100 dilution, CST) overnight at 4 °C. The PV-9000 polymer detection system was employed: reaction-boosting solution (reagent 2) and HRP-conjugated secondary antibody (reagent 3) were applied sequentially (20 min each, room temperature). Chromogenic development was performed using DAB (5 min; Beyotime Biotechnology) with hematoxylin counterstaining and permanent mounting with neutral resin. Whole-slide imaging was performed using a Panoramic MIDI scanner (Jetta JD801 system, Jiangsu Jetta Technology). MET expression was semiquantitatively assessed by combined intensity/distribution scoring, with subsequent stratification into low (lower median) and high (upper median) expression cohorts for comparative analysis.

### Cell culture and transfection

The human thyroid cancer cell lines BCPAP and TPC1 were acquired from the American Type Culture Collection (ATCC, Manassas, VA, USA) and routinely screened to maintain mycoplasma-free status. *MET* knockout lentiviruses were used to generate BCPAP/*MET*^−/−^, and TPC1/*MET*^−/−^ according to the instructions from the manufacturer (Sangon Biotech (Shanghai) Co., Ltd.). The c-MET-targeting shRNA sequences used for lentiviral knockdown were as follows: pLVE3163(CCGGGCTGTGAGAATATACACTTACCTCGAGGTAAGTGTATATTCTCACAGCTTTTTTG), pLVE3164(CCGGCCTTCAGAAGGTTGCTGAGTACTCGAGTACTCAGCAACCTTCTGAAGGTTTTTTG), and pLVE3165(CCGGTCAACTTCTTTGTAGGCAATACTCGAGTATTGCCTACAAAGAAGTTGATTTTTTG). Thyroid cancer cells were infected with the lentiviruses at approximately 50% confluency. After 48 h, transfection efficiency was assessed via microscopy, and *MET* knockdown efficiency was verified by Western blot analysis. BCPAP and TPC1 cells were maintained in complete RPMI 1640 (HyClone, UT, USA) and DMEM (HyClone, UT, USA) media, respectively, supplemented with 10% fetal bovine serum (FBS; ExCell Bio, Suzhou, China) and 1% penicillin–streptomycin solution (Solarbio, Beijing, China). All cell cultures were incubated at 37 °C in a humidified 5% CO_2_ incubator.

### Western blotting (WB)

Western blotting was performed as previously described^38^. Total protein lysates were separated on 10% SDS-polyacrylamide gels and transferred onto polyvinylidene difluoride (PVDF) membranes (Millipore, Billerica, MA, USA) using a wet transfer apparatus. For immunodetection, membranes were incubated with the following primary antibodies: anti-GAPDH (Duoneng Biotechnology; 1:1000, loading control), anti-MET (CST; 1:1000), anti-ERK(CST; 1:1000), anti-p-ERK(CST; 1:1000), anti-STAT3 (CST; 1:1000), anti-p-STAT3 (CST; 1:1000), anti-FN1(CST; 1:1000), anti-MMP2(Abcam; 1:1000), anti-MMP7(Abcam; 1:1000), anti-MMP9(Abcam; 1:1000), and anti-Twist1(CST; 1:1000). After extensive washing, membranes were probed with appropriate HRP-conjugated secondary antibodies (Cell Signaling Technology; 1:2000) for 1 h at room temperature. Immunoreactive bands were detected using ECL substrate and visualized with a chemiluminescence imaging system (Clinx Science Instruments, Shanghai, China). Band intensities were quantified using ImageJ v.1.44p.

### Wound healing assay

Wound healing assays were performed by plating cells in 12-well plates until reaching 90–100% confluence. Linear wounds were created in the monolayer using a sterile 10 µL pipette tip, maintaining uniform wound widths across replicates. Cell migration was monitored at baseline (0 h) and 24 h post-wounding using an inverted microscope. The wound closure rate was calculated as: Migration rate = [(W_0_ − W_24_)/W_0_] × 100, where W_0_ and W_24_ represent wound widths at 0 h and 24 h, respectively.

### Transwell assay

Cell migration was evaluated using a 24-well Boyden chamber (Corning, Cat# 3422, NY, USA). Cells were harvested and adjusted to 5 × 10^4^ cells/mL in serum-free medium, and 250 µL aliquots were seeded in the upper chamber. The lower chamber contained 750 µL of complete medium supplemented with 20% FBS as a chemoattractant. After 24 h incubation at 37 °C, non-migratory cells were removed from the upper chamber using cotton swabs. Membrane-adherent cells were fixed with methanol (20 min), stained with crystal violet (15 min), and quantified under an inverted microscope.

### Immune cell infiltration analysis

The association between MET expression patterns and immune cell infiltration in THCA was analyzed using the Tumor Immune Estimation Resource (TIMER, https://cistrome.shinyapps.io/timer/). This validated bioinformatics platform enables comprehensive profiling of immune cell abundances and their molecular correlations across diverse malignancies. Specifically, we evaluated infiltration levels of five major immune subsets: B cells, CD4^+^ T cells, CD8^+^ T cells, neutrophils, and macrophages.

### Statistical analysis

All statistical analyses were performed using GraphPad Prism 8.4.0. Intergroup differences were assessed using unpaired Student’s t-tests for comparisons between two groups or one-way ANOVA for multiple group comparisons. Survival analysis was conducted using the Kaplan–Meier method with log-rank testing. To evaluate associations between variables, we utilized Spearman’s rank correlation analysis. A two-tailed P-value < 0.05 was considered statistically significant.

## Conclusions

This study provides a comprehensive elucidation of MET’s oncogenic role in THCA progression through multi-modal investigations. Bioinformatics profiling demonstrated that MET overexpression in THCA is associated with shorter survival and aggressive metastatic behavior, observations corroborated by immunohistochemical analysis of clinical samples. Functional characterization revealed that MET knockdown substantially attenuates THCA cell migration, mediated through ERK/STAT3 signaling-dependent modulation of metastatic effectors. Notably, we established novel associations between MET overexpression and both loss of thyroid differentiation markers and altered immune cell infiltration patterns. Collectively, these findings establish MET as a multifaceted regulator of THCA pathogenesis, offering compelling evidence for pursuing MET-targeted therapies in advanced disease.

## Supplementary Information

Below is the link to the electronic supplementary material.

Supplementary Material 1

Supplementary Material 2

## Data Availability

The authors confirm that the data supporting the findings of this study are available within the article and its supplementary materials.

## Abbreviations

THCA: Thyroid cancer
GSEA: Gene set enrichment analysis
WB: Western blotting
IHC: Immunohistochemistry
