# Supplementary material for: Overexpressed MET drives aggressive thyroid cancer phenotypes and serves as a precision therapeutic target
Source: Sci Rep. 2025 Nov 13;15:39809. doi: 10.1038/s41598-025-23587-7 (PMC12615610; doi:10.1038/s41598-025-23587-7)

F1D-Patient1-Normal

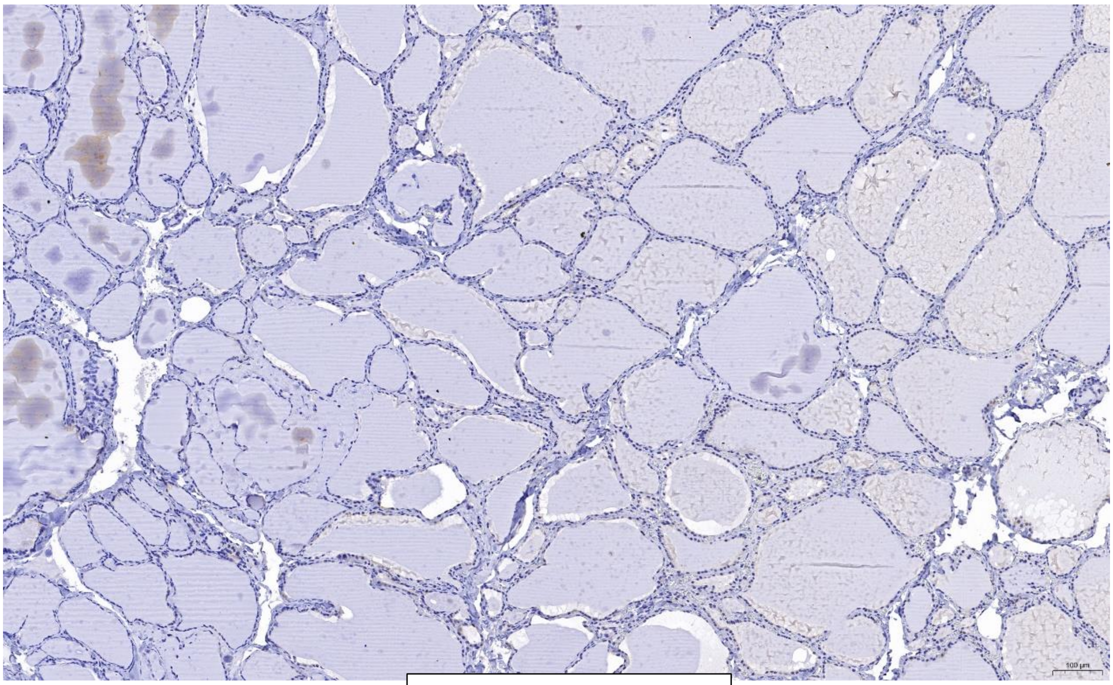

F1D-Patient1-Tumor

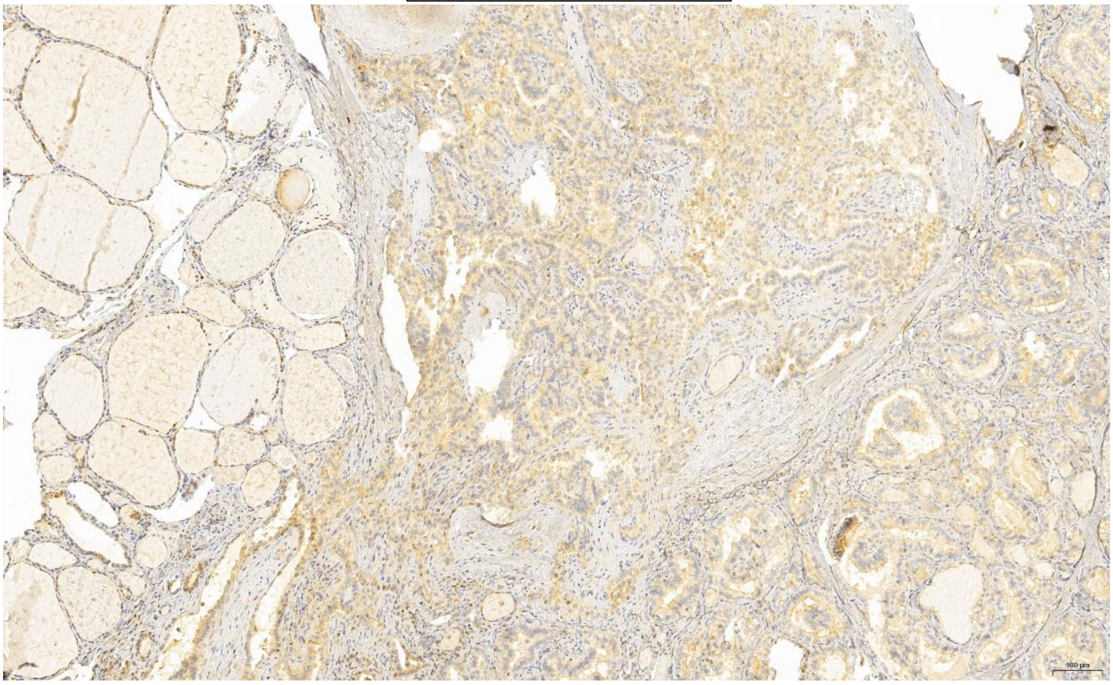

F1D-Patient2-Normal

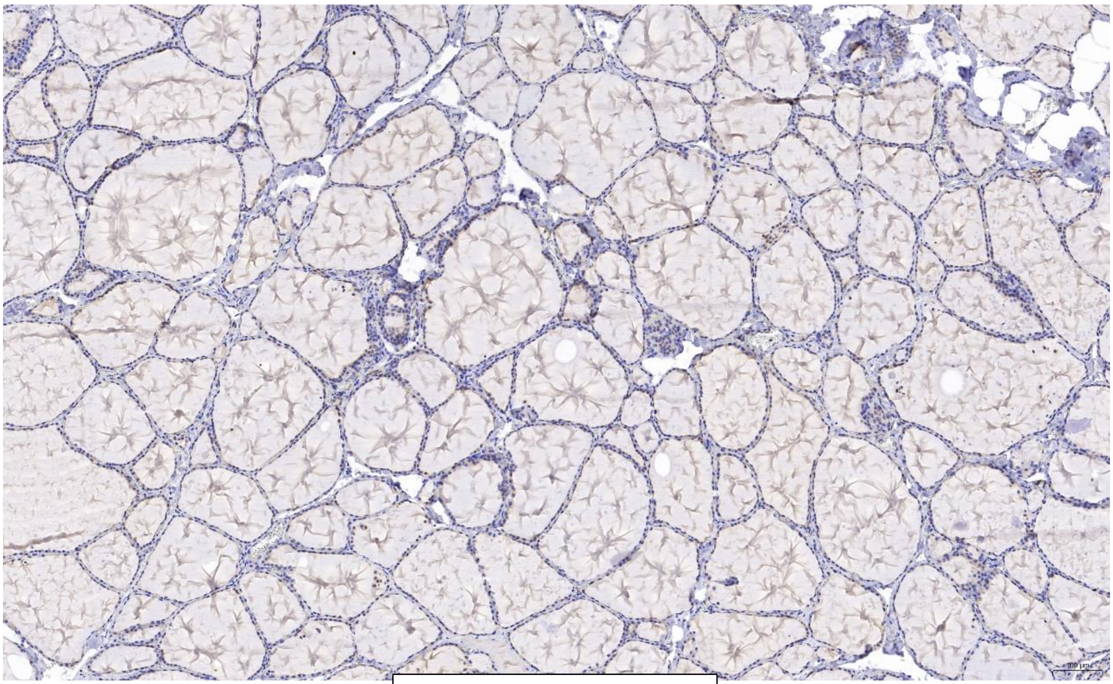

F1D-Patient2-Tumor

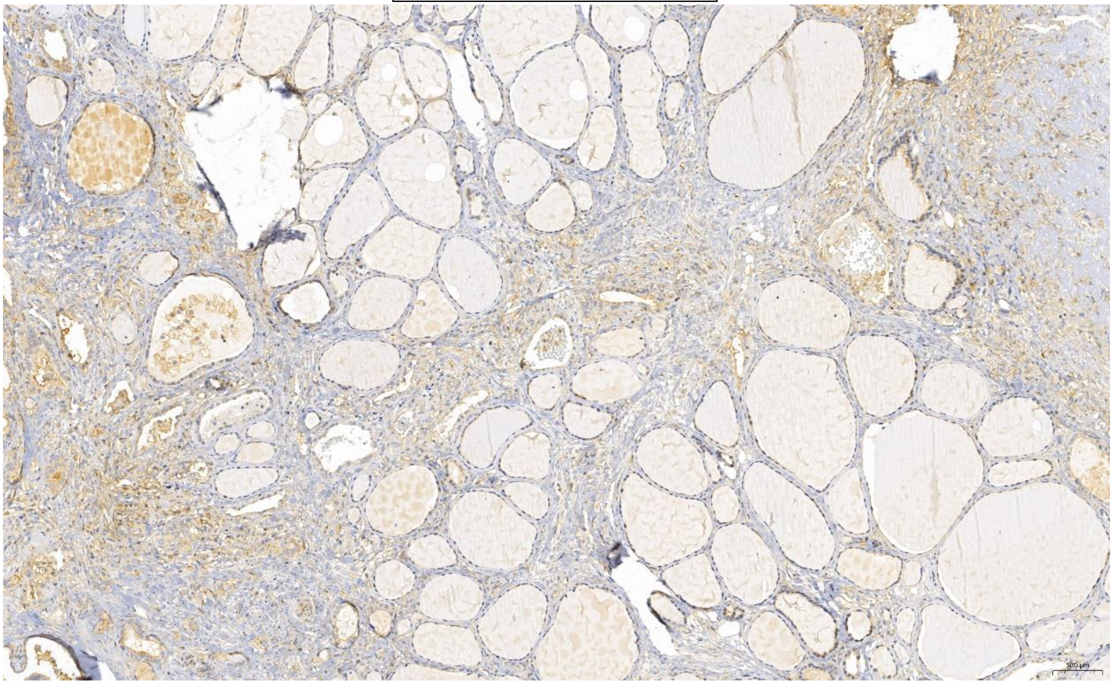

F1D-Patient3-Normal

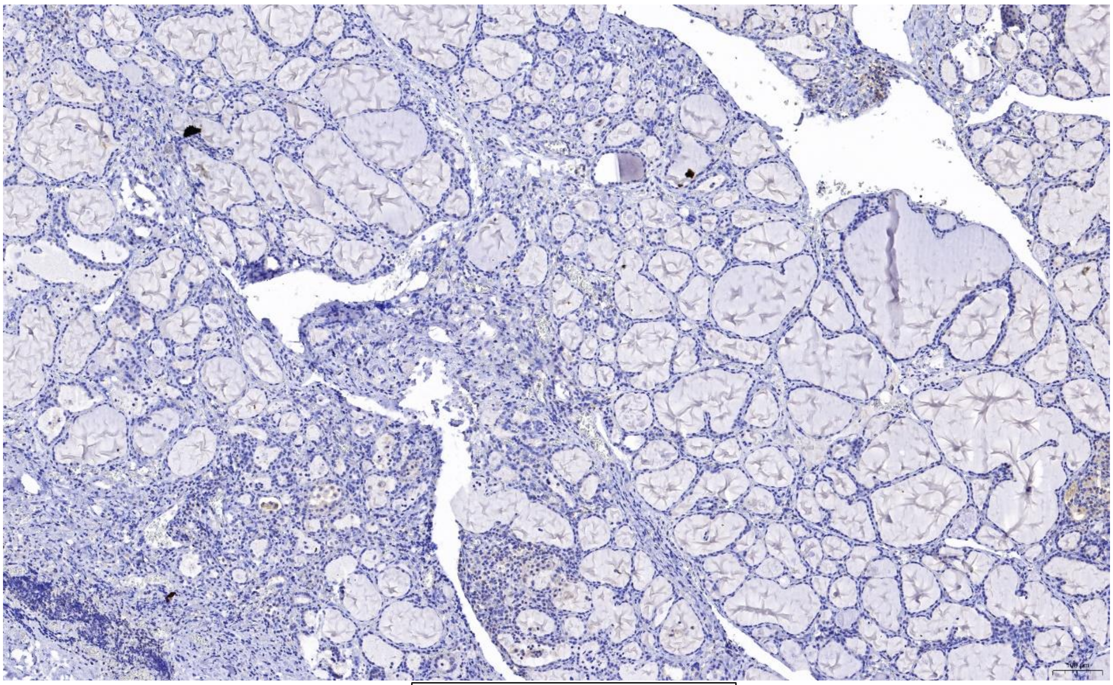

F1D-Patient3-Tumor

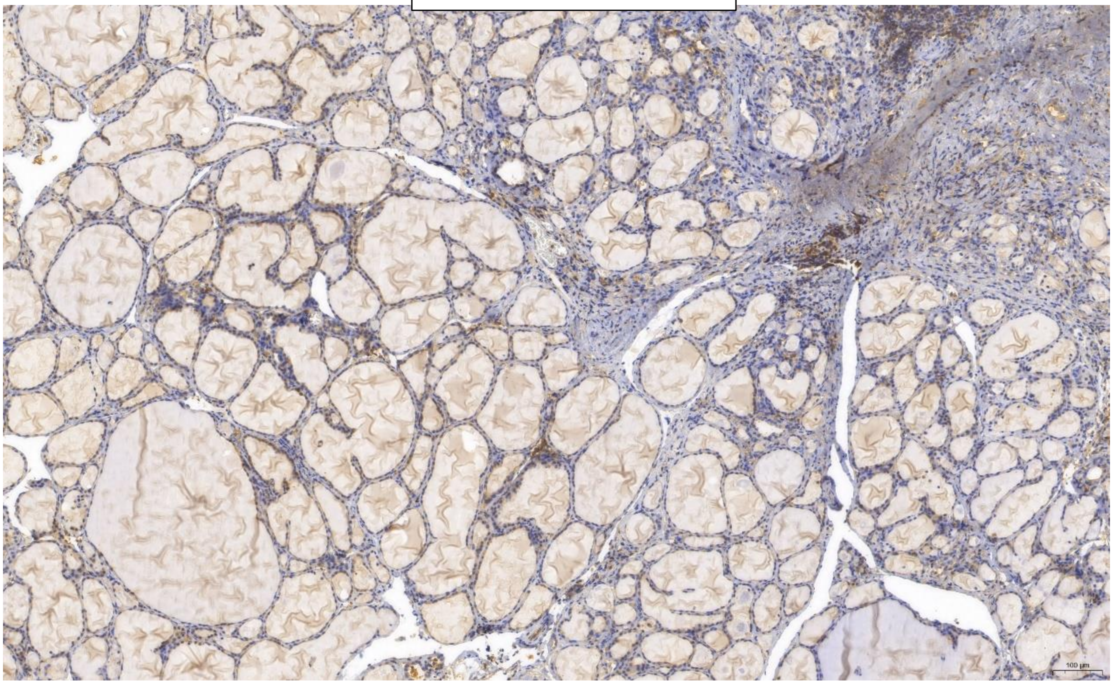

F1D-Patient4-Normal

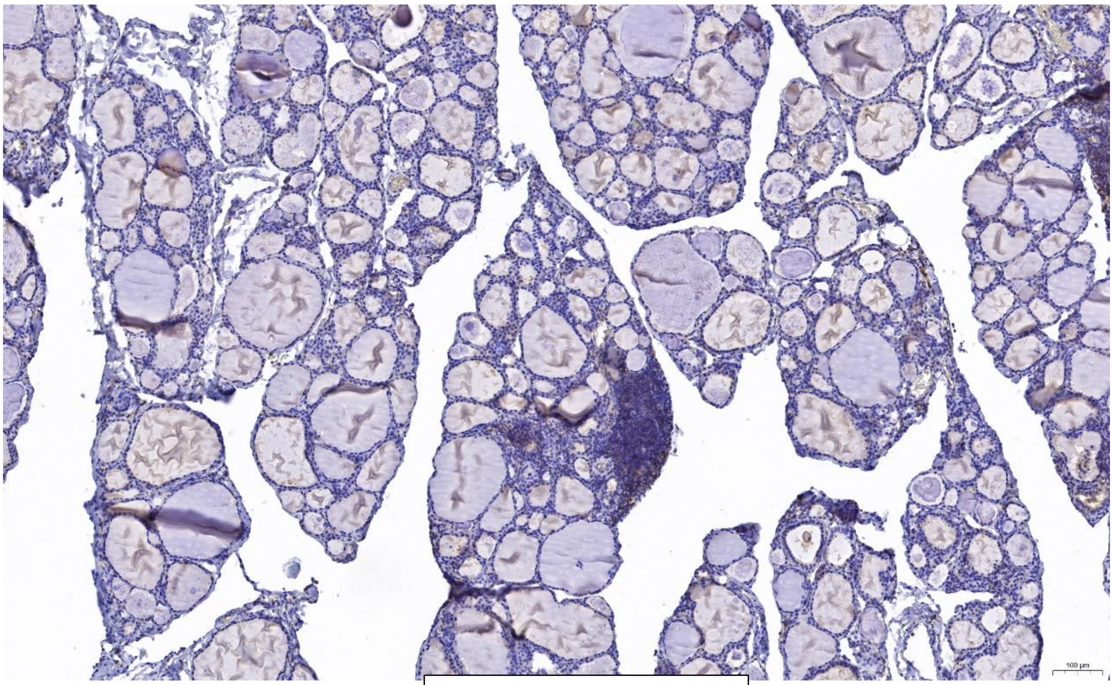

F1D-Patient4-Tumor

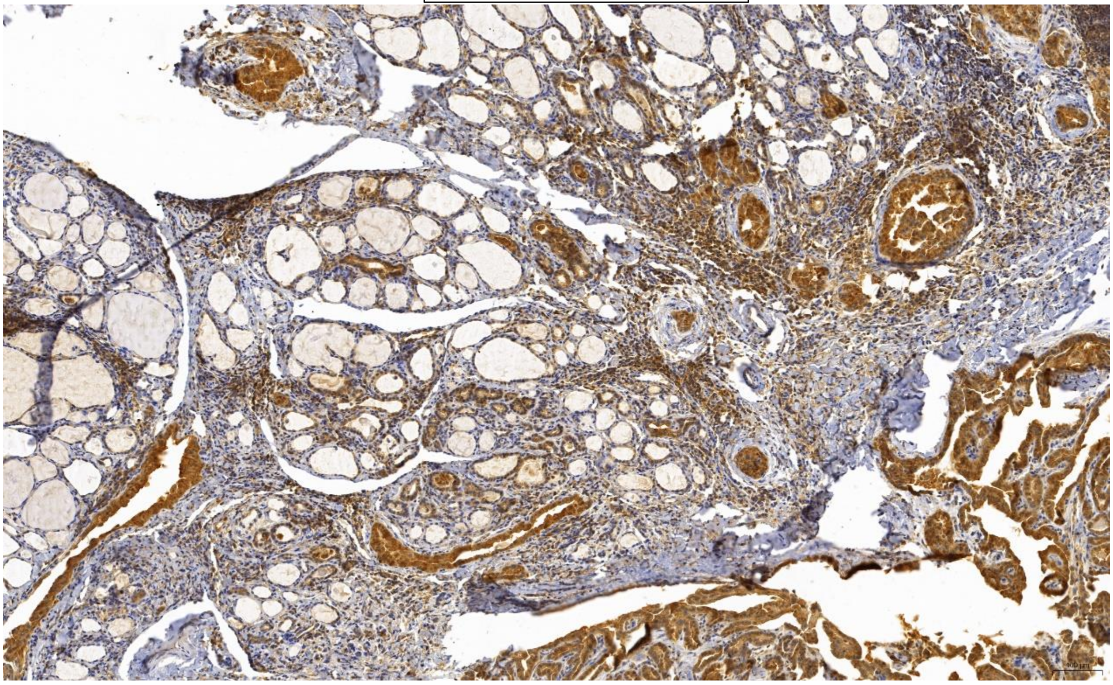

F1D-Patient5-Normal

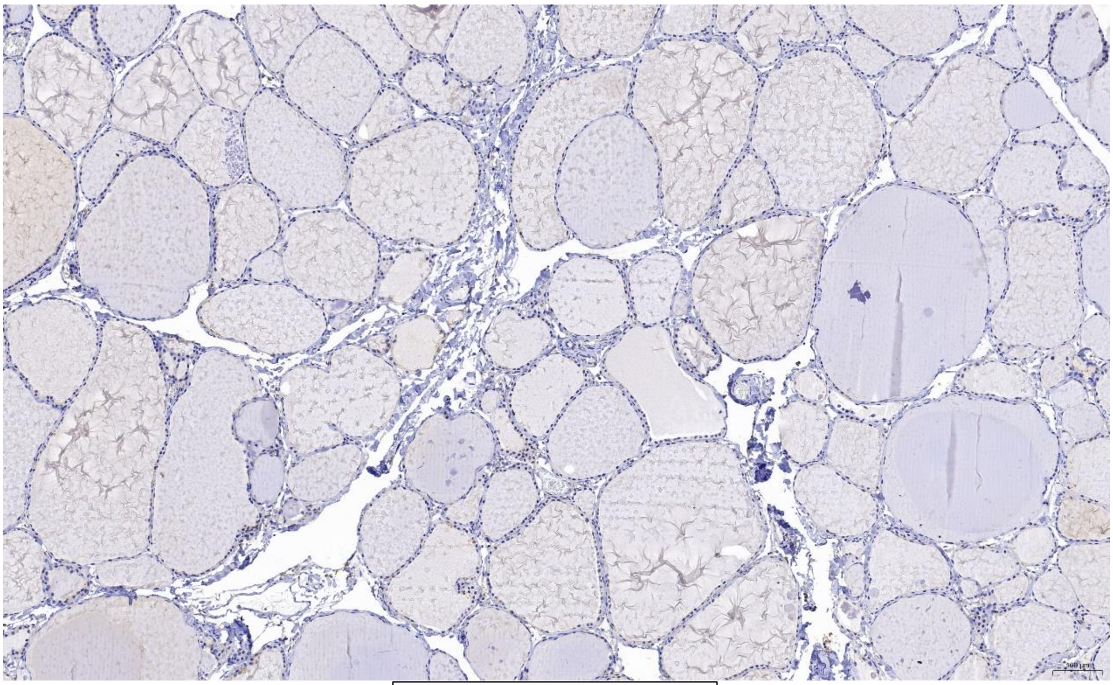

F1D-Patient5-Tumor

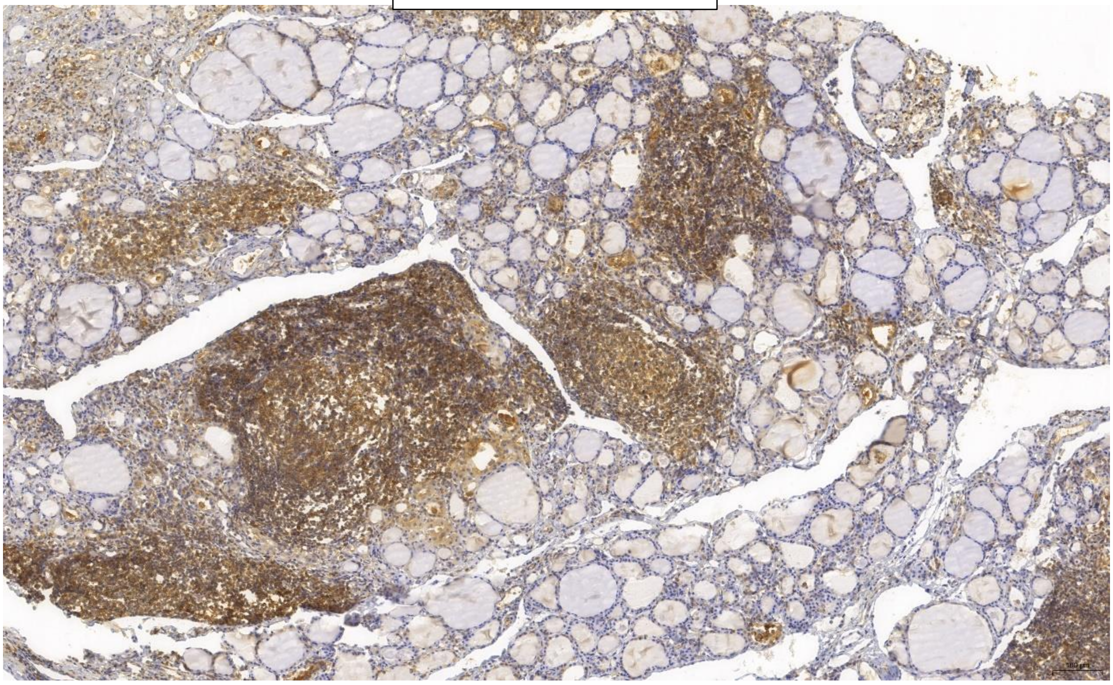

F1D-Patient6-Normal

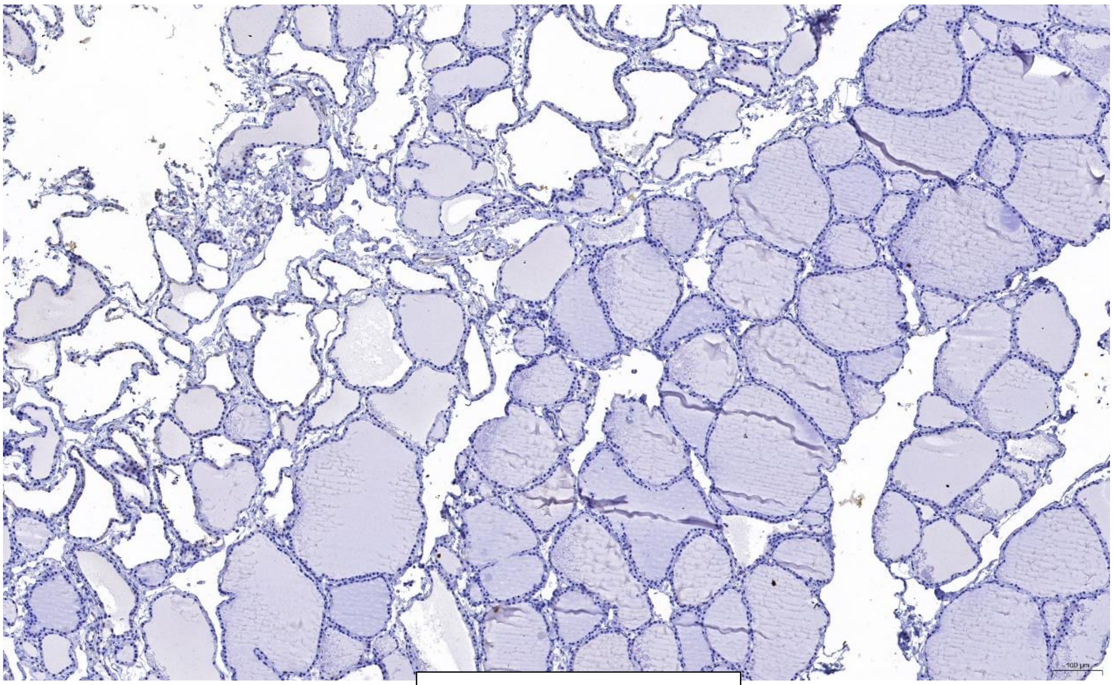

F1D-Patient6-Tumor

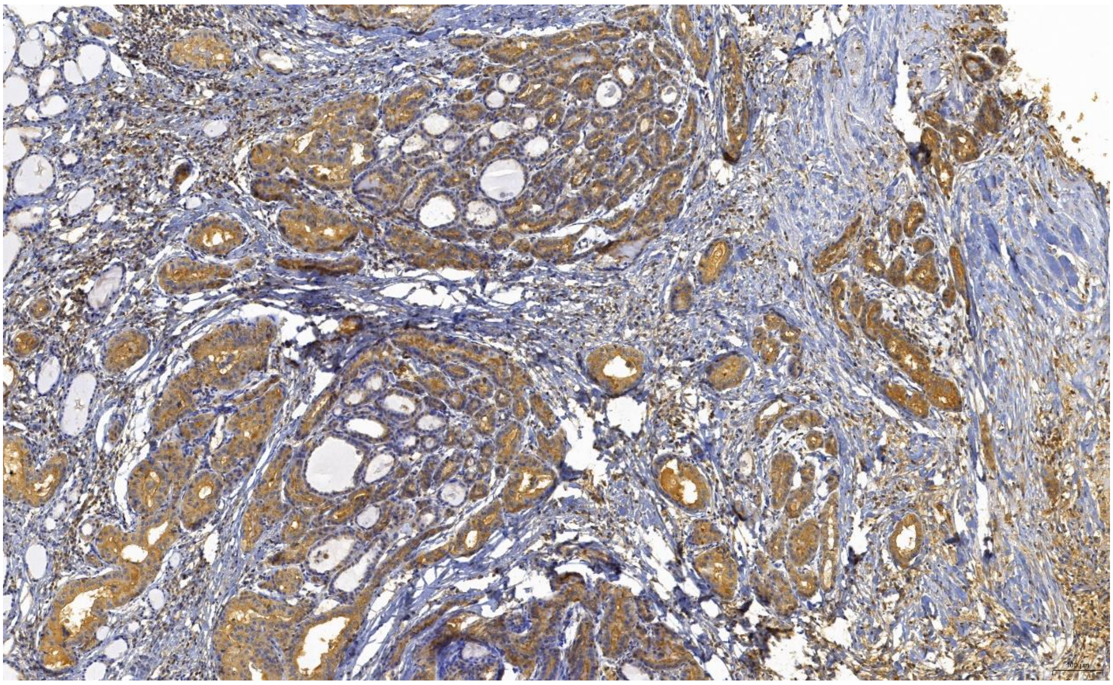

F2E-Patient7-Normal

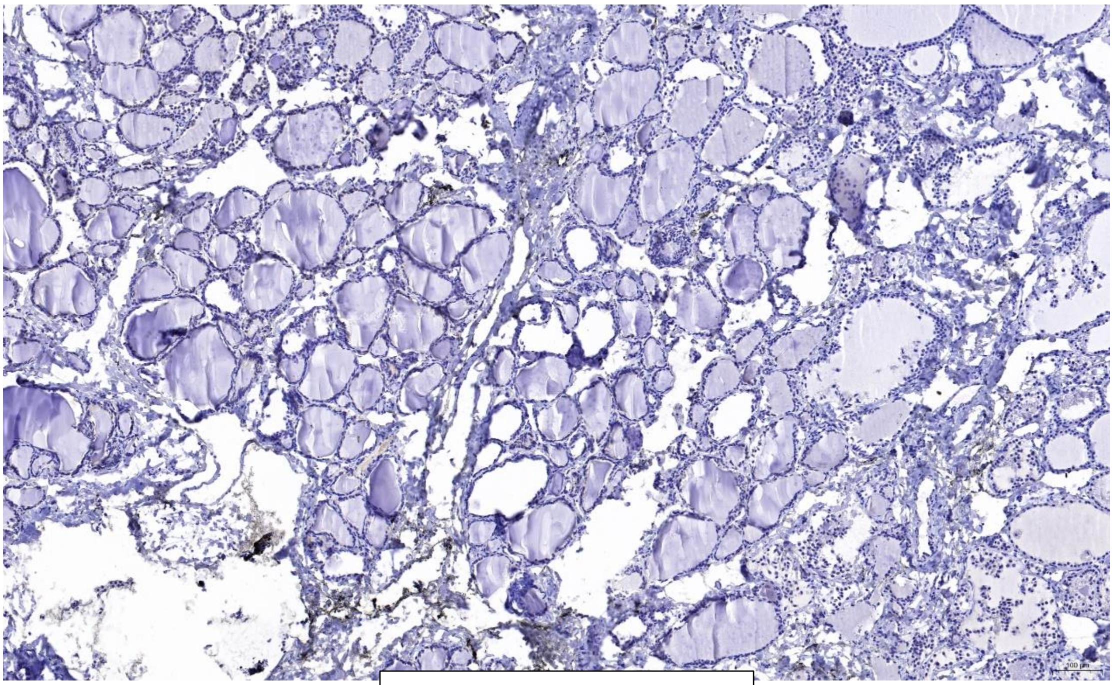

F2E-Patient7-Normal-HE

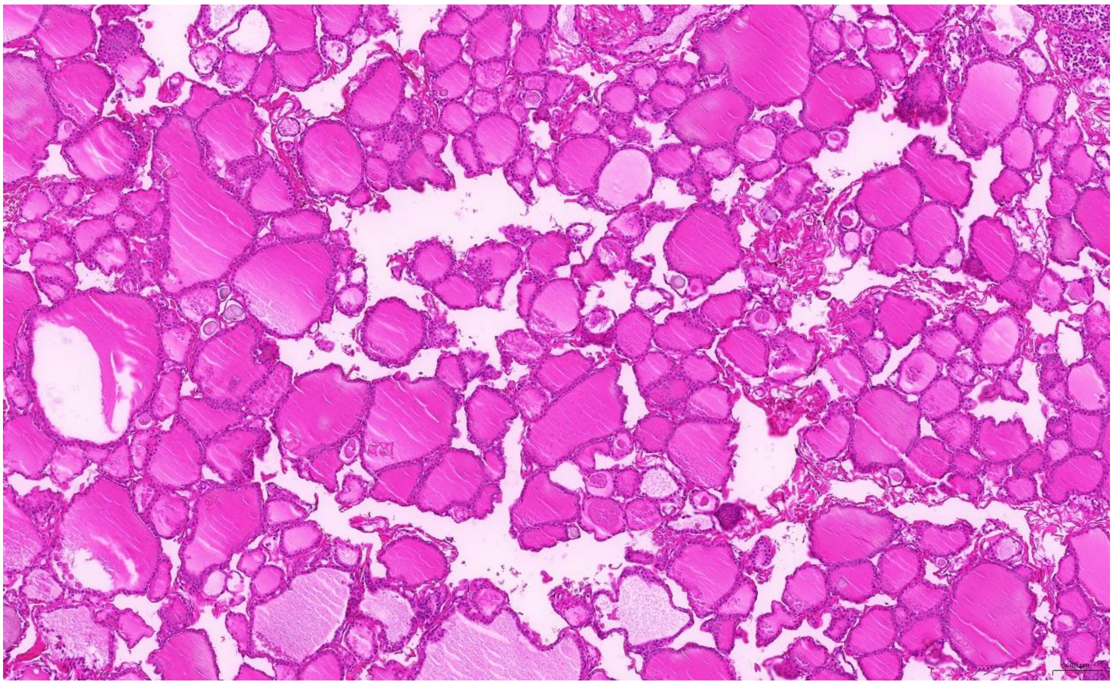

F2E-Patient7-Tumor

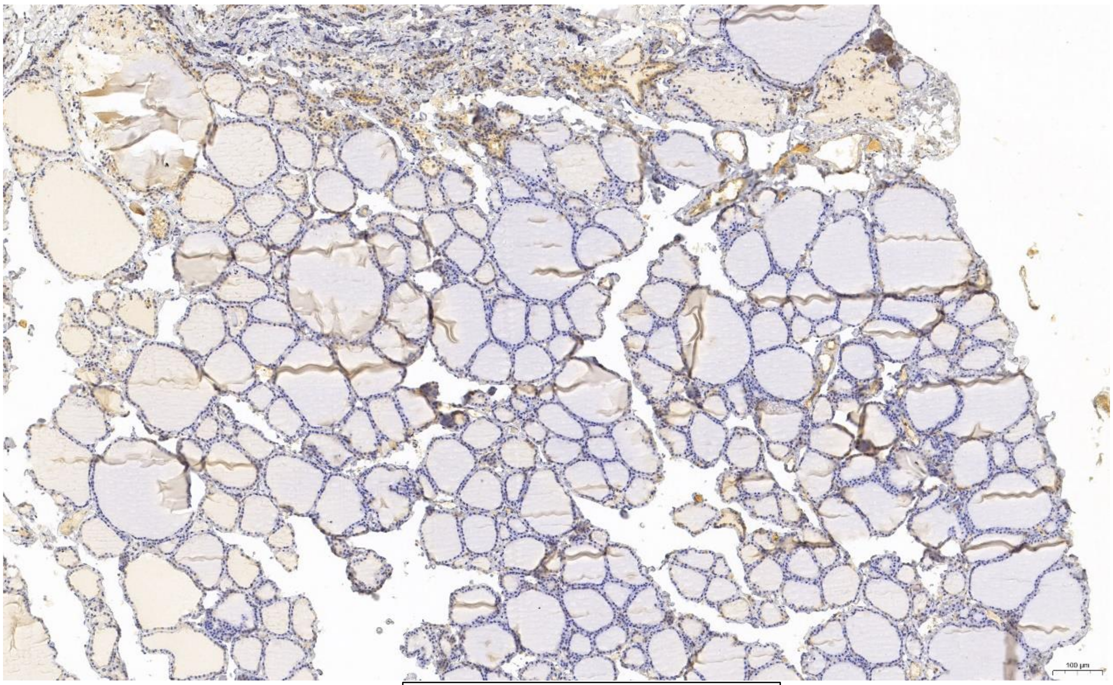

F2E-Patient7-Tumor-HE

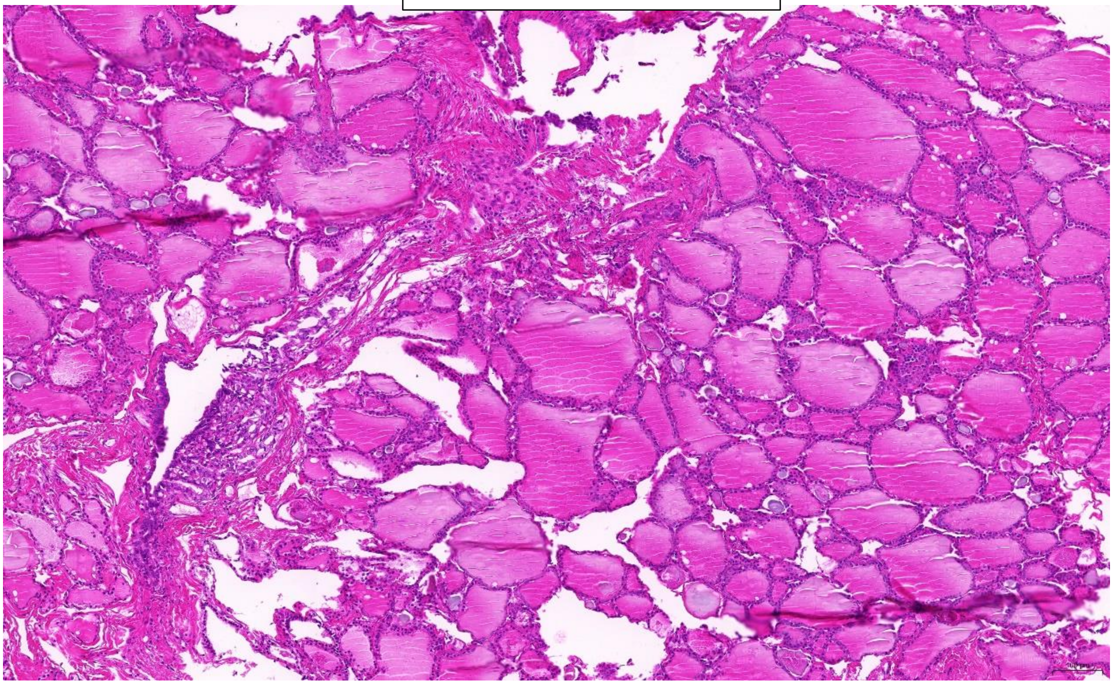

F2E-Patient8-Normal

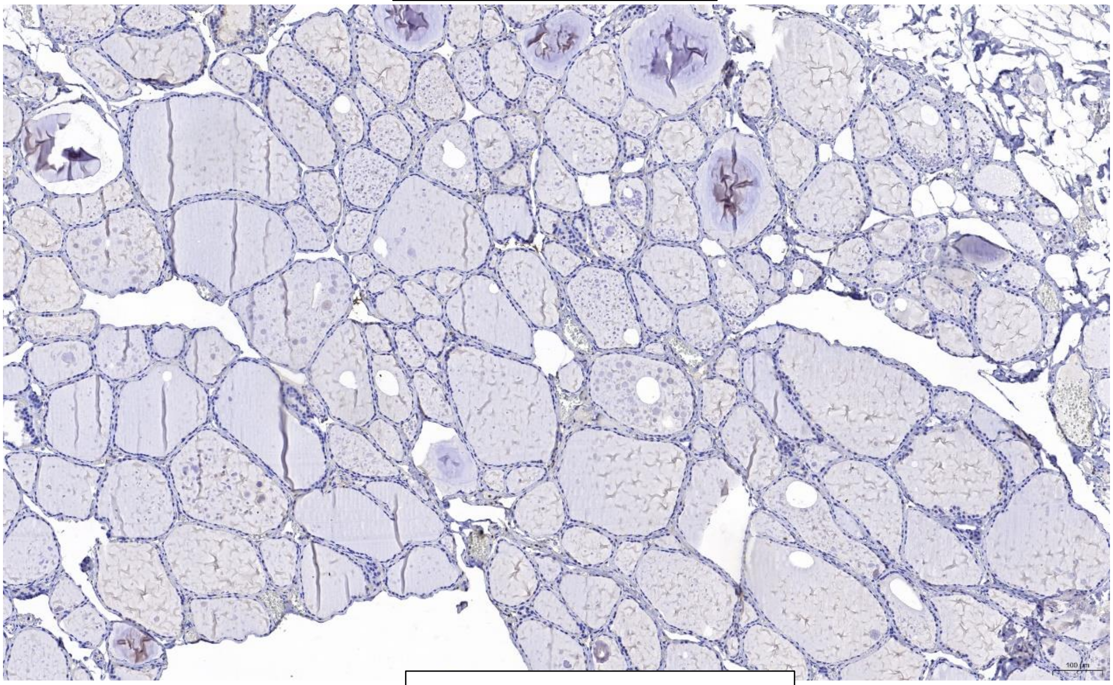

F2E-Patient8-Normal-HE

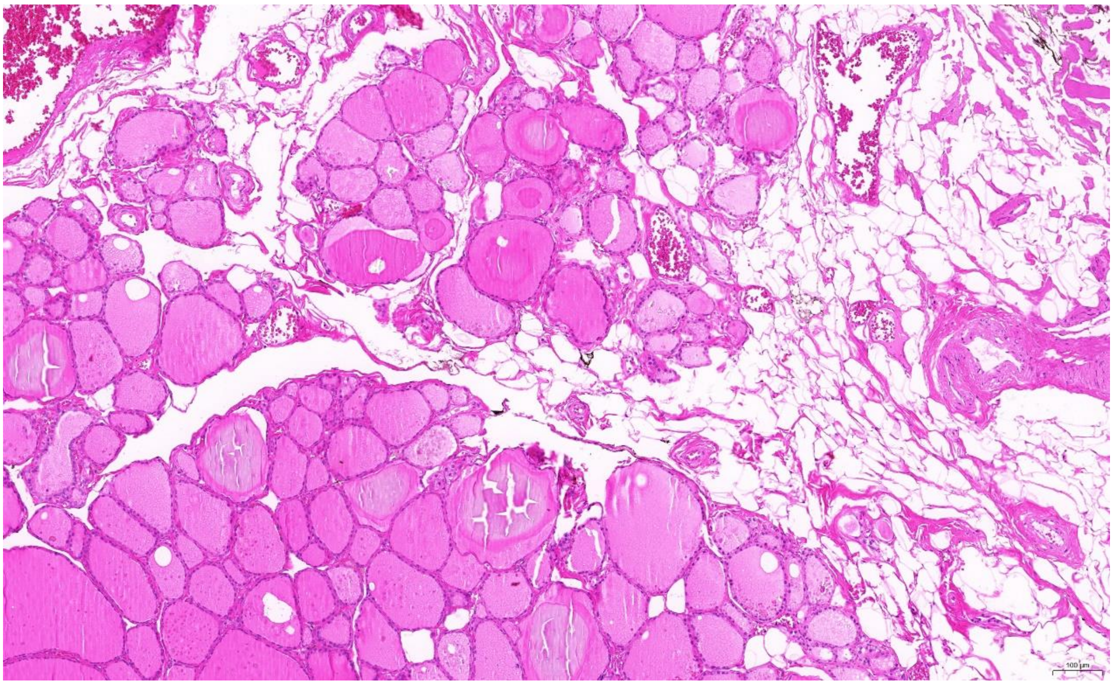

F2E-Patient8-Tumor

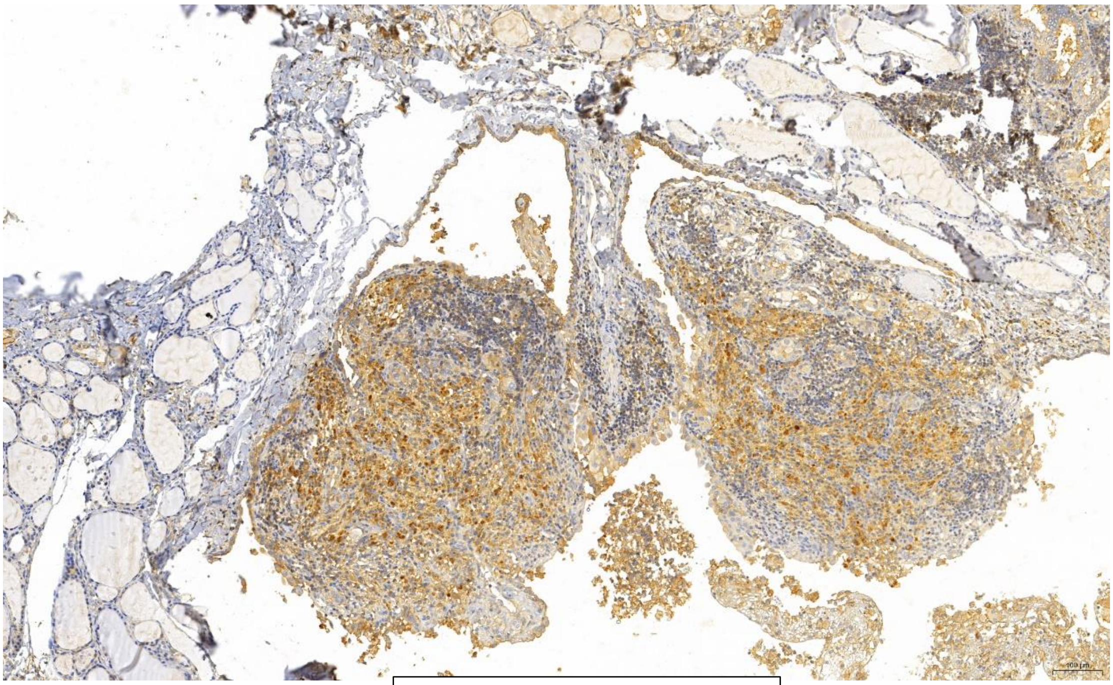

F2E-Patient8-Tumor-HE

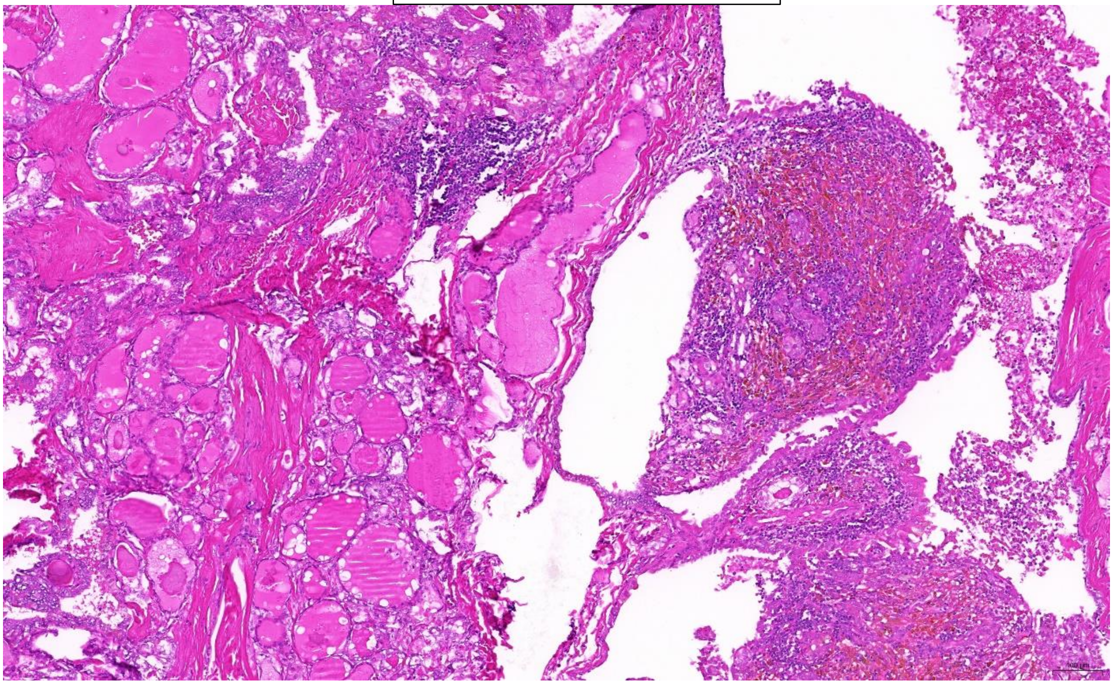

F2E-Patient9-Normal

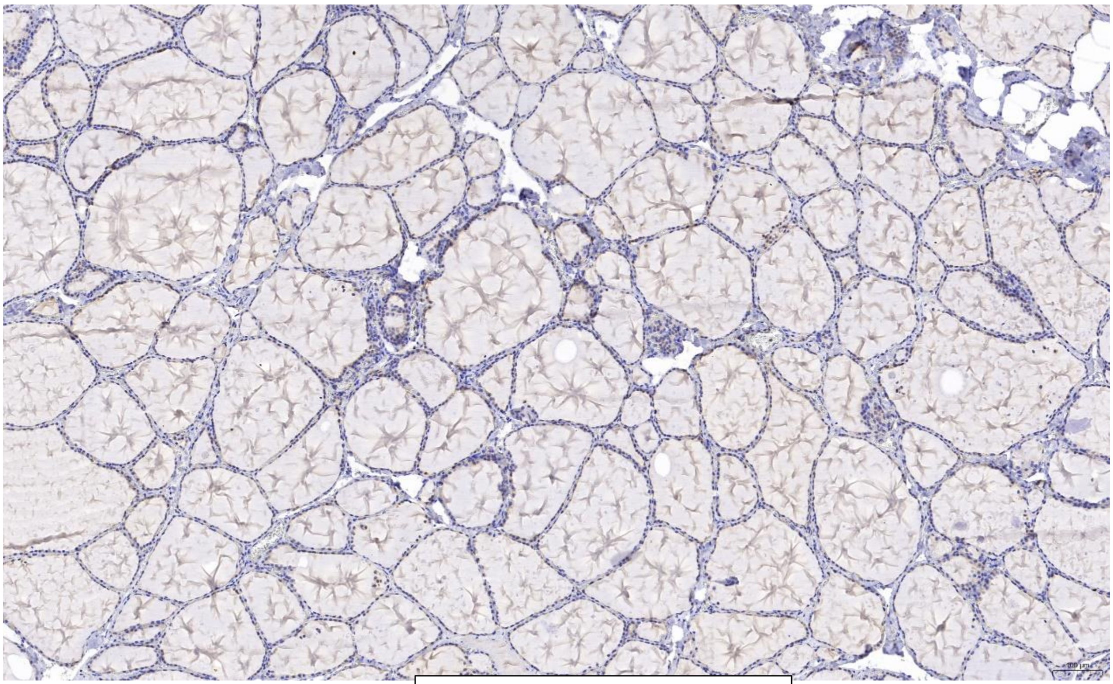

F2E-Patient9-Normal-HE

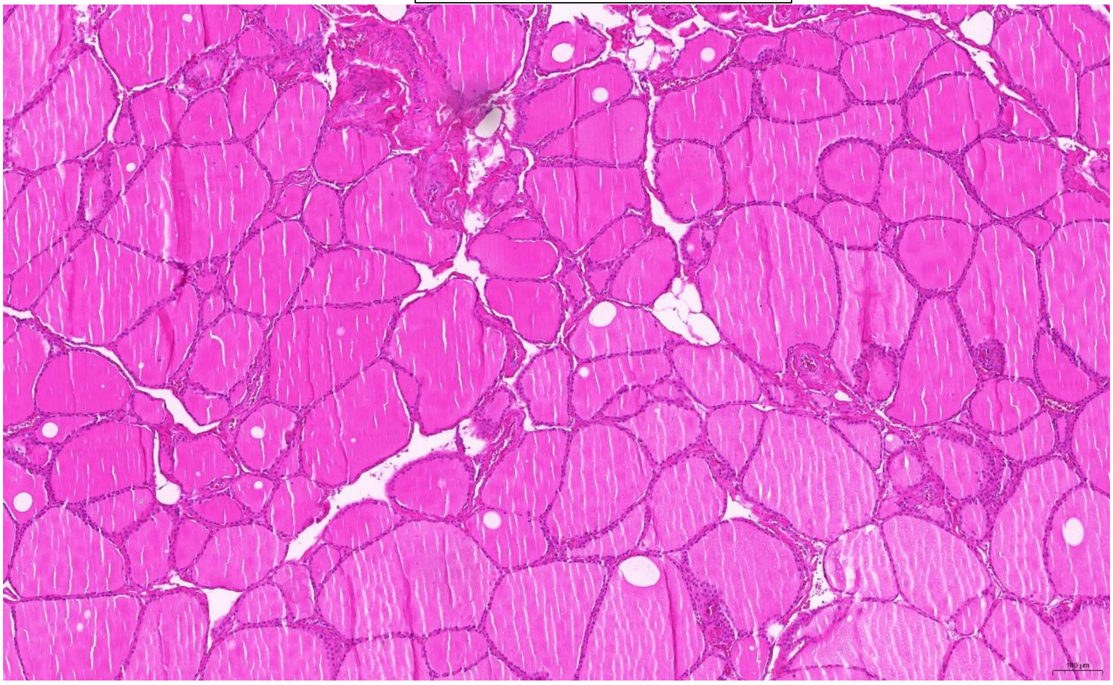

F2E-Patient9-Tumor

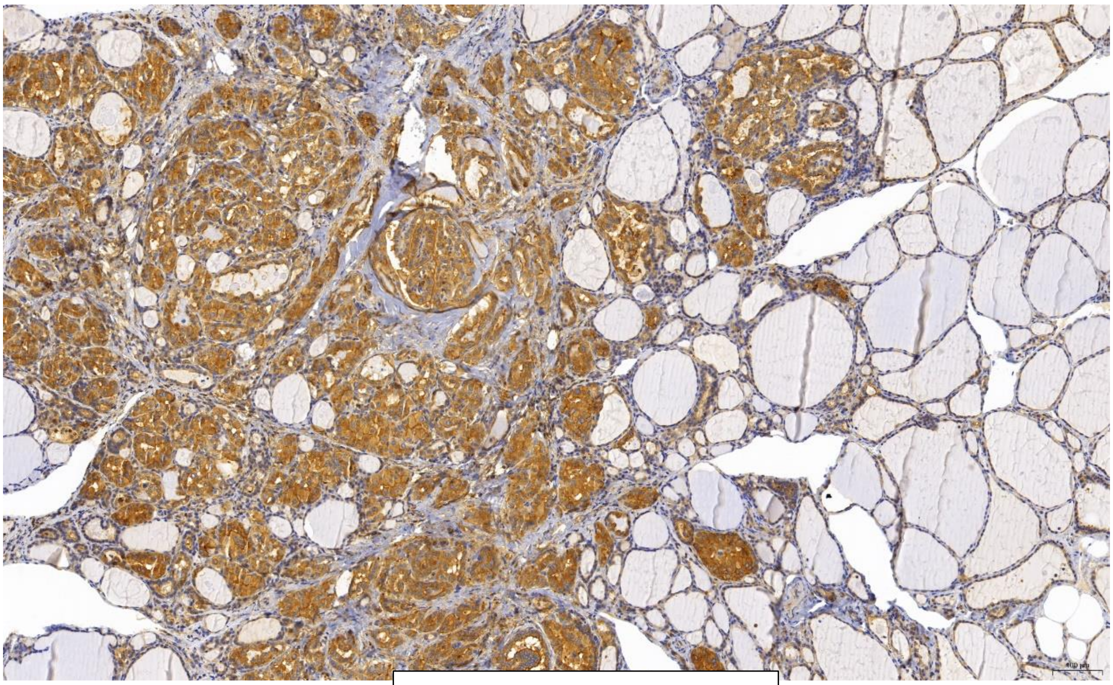

F2E-Patient9-Tumor-HE

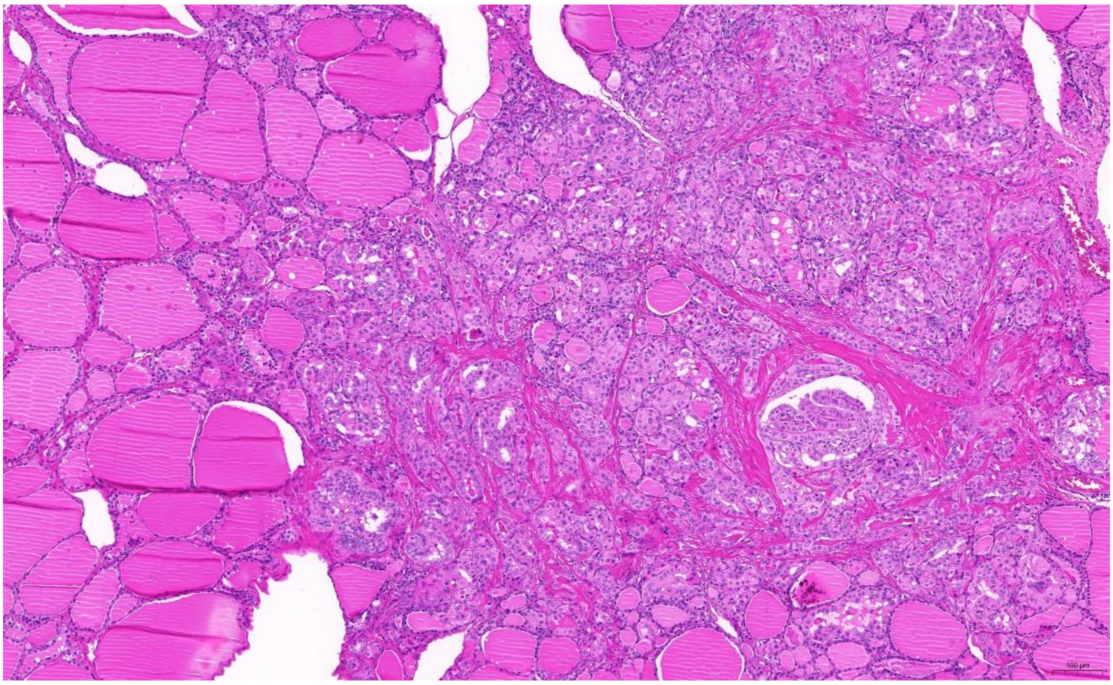

F3C-BCPAP-0h

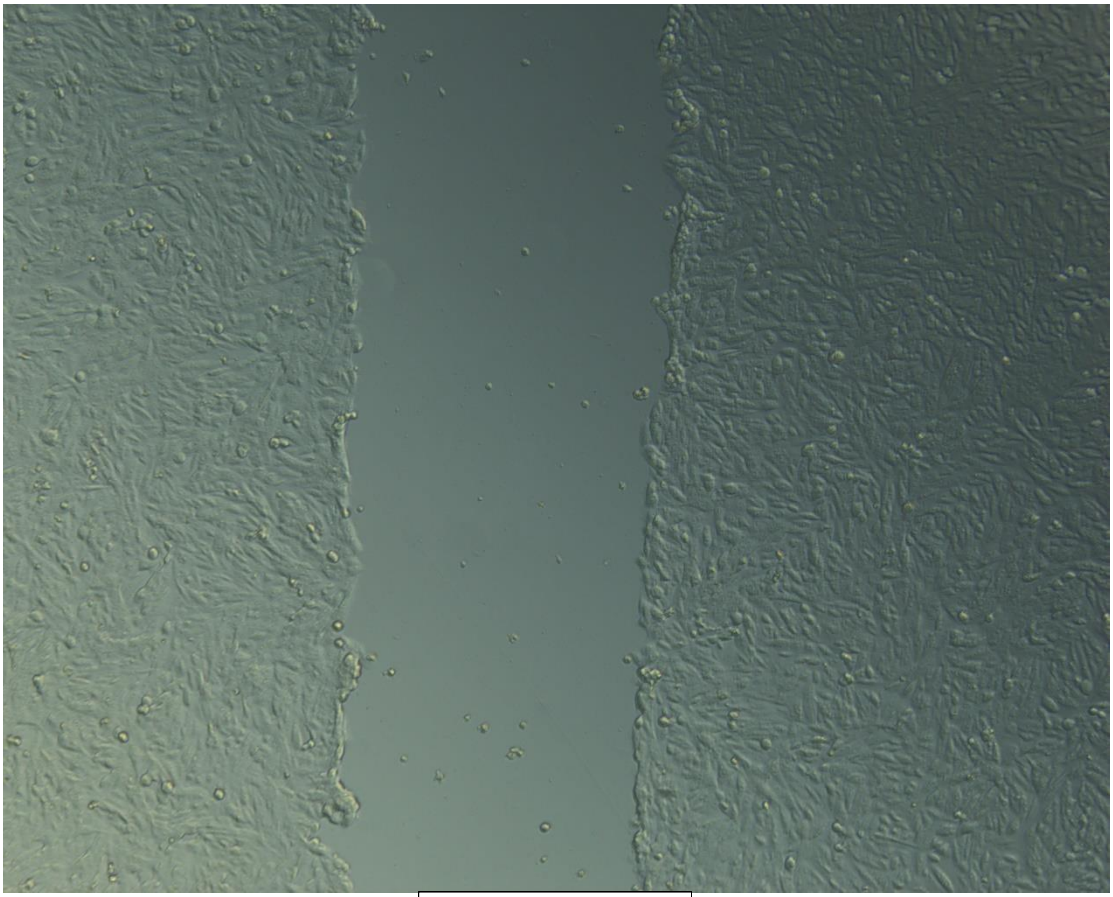

F3C-BCPAP-24h

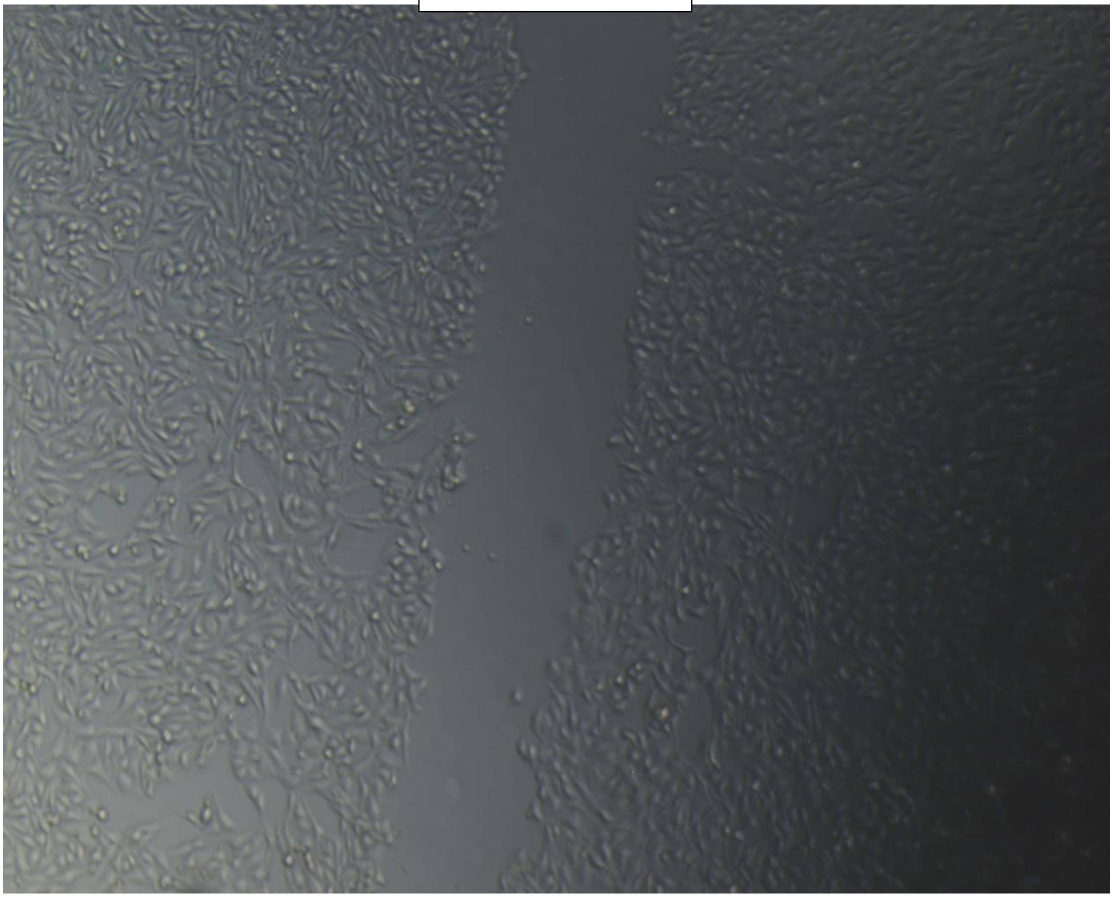

F3C-BCPAP$^{Met-/-}$-0h

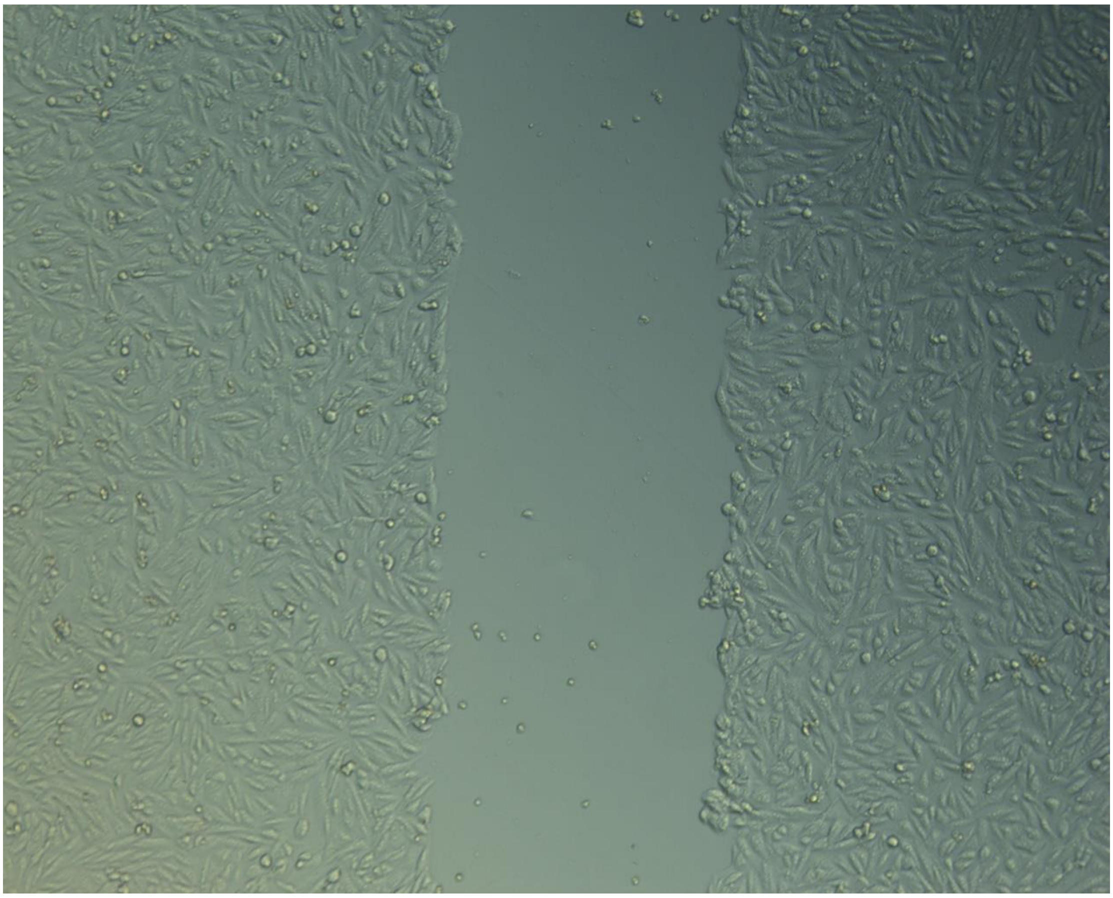

F3C-BCPAP$^{Met-/-}$-24h

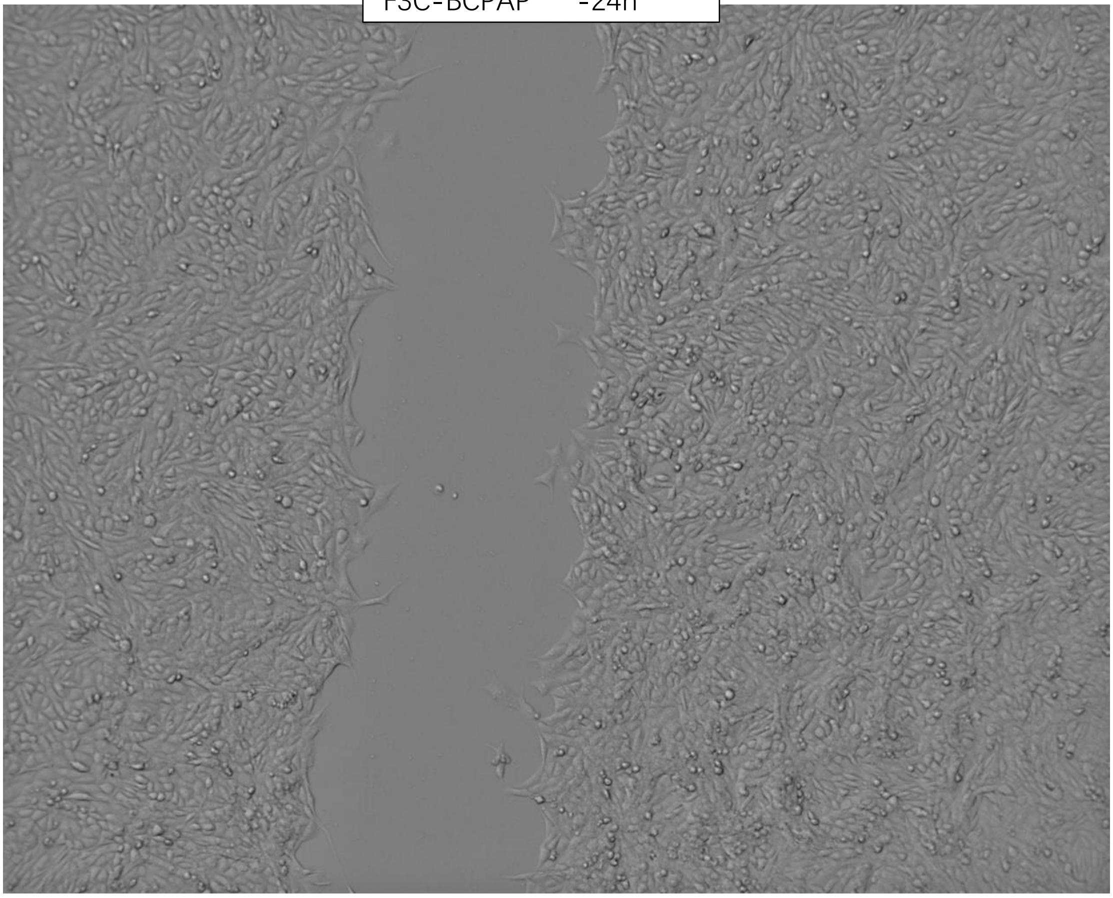

F3C-TPC1-0h

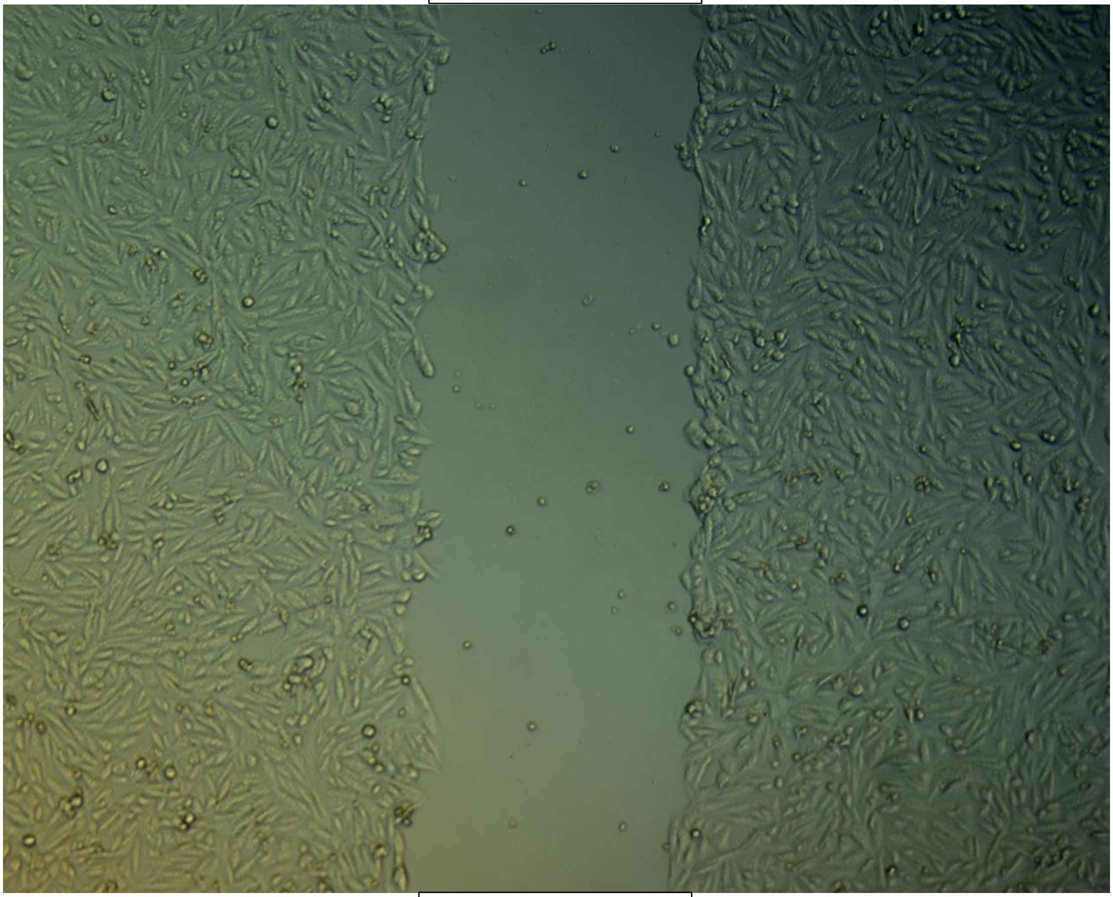

F3C-TPC1-24h

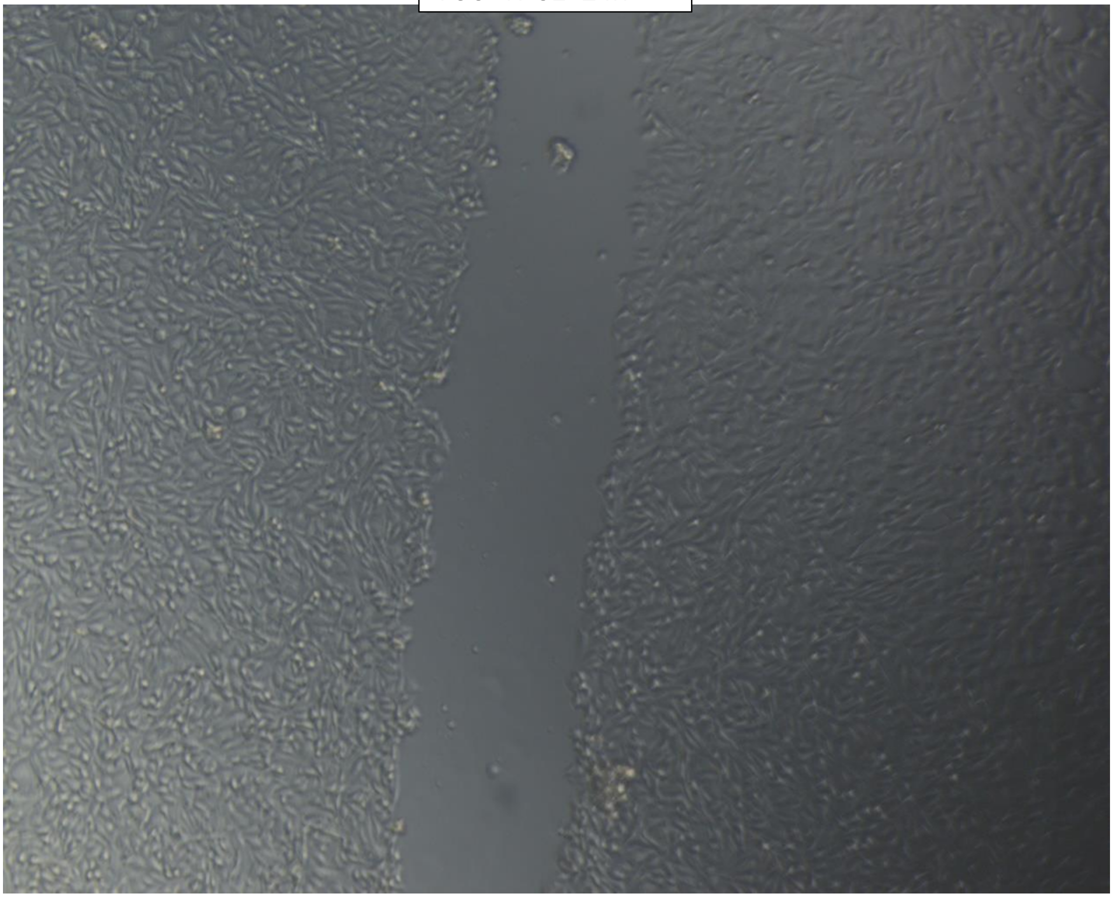

F3C-TPC1$^{Met-/-}$-0h

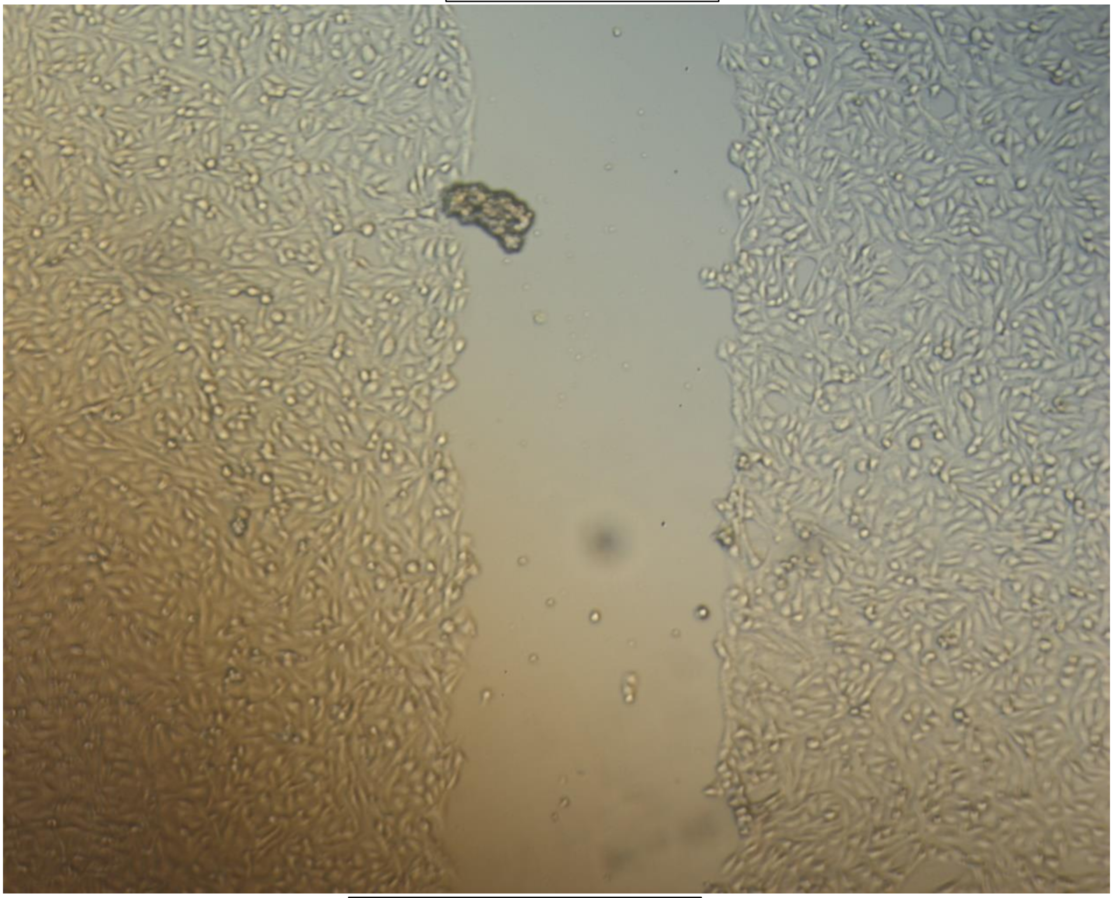

F3C-TPC1$^{Met-/-}$-24h

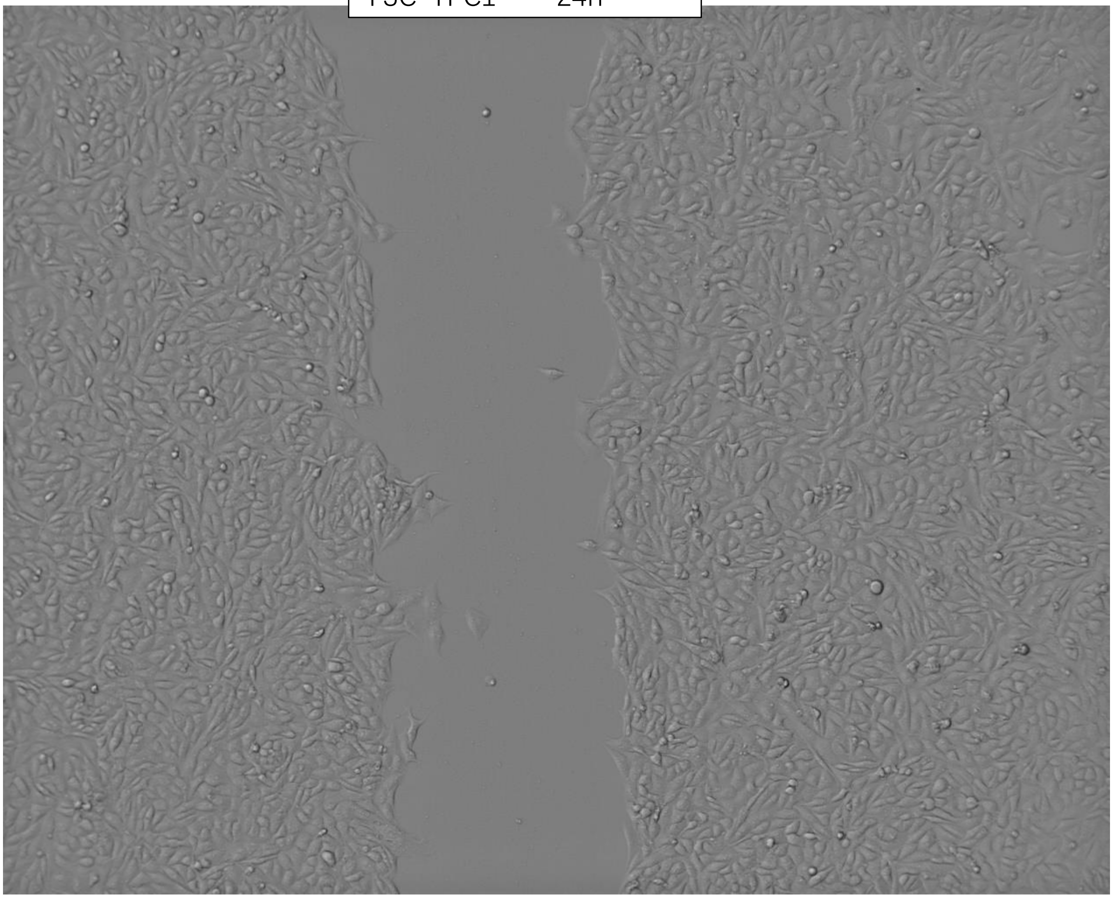

F3D-BCPAP$^{Met-/-}$

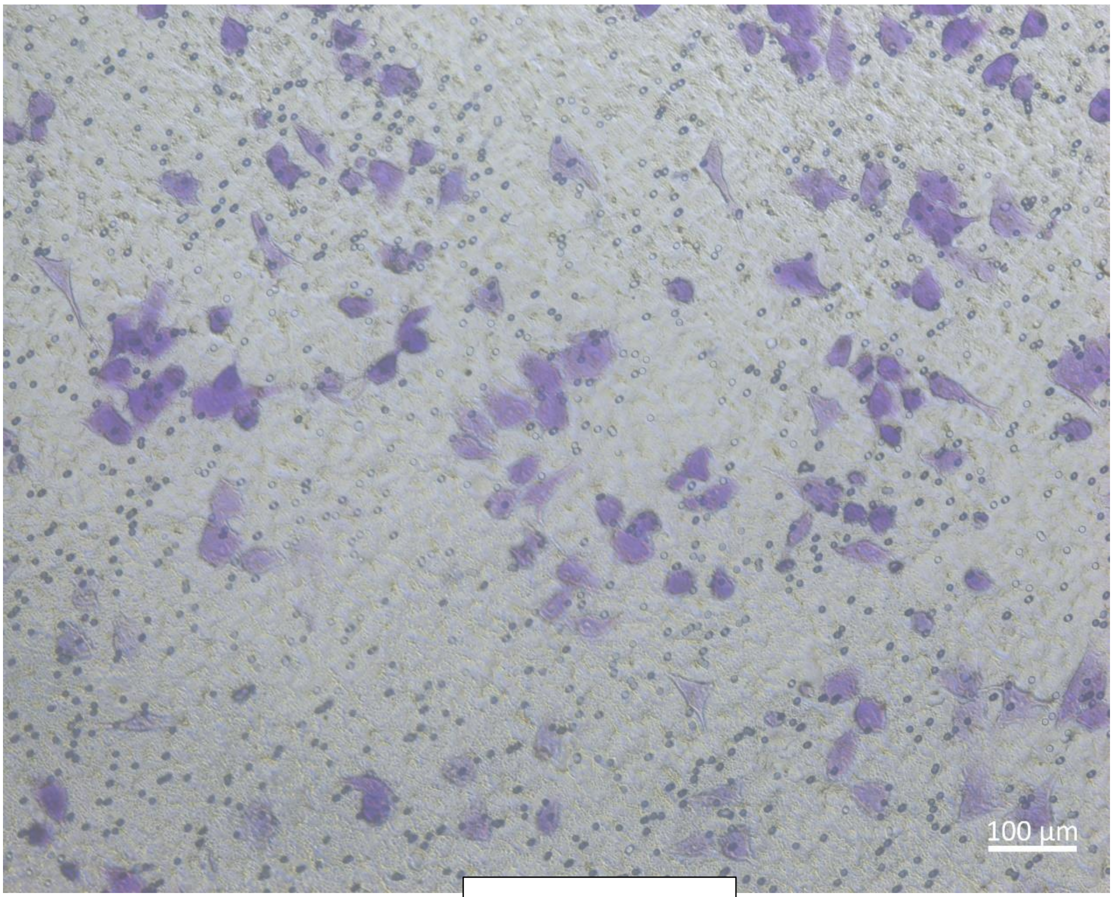

F3D-BCPAP

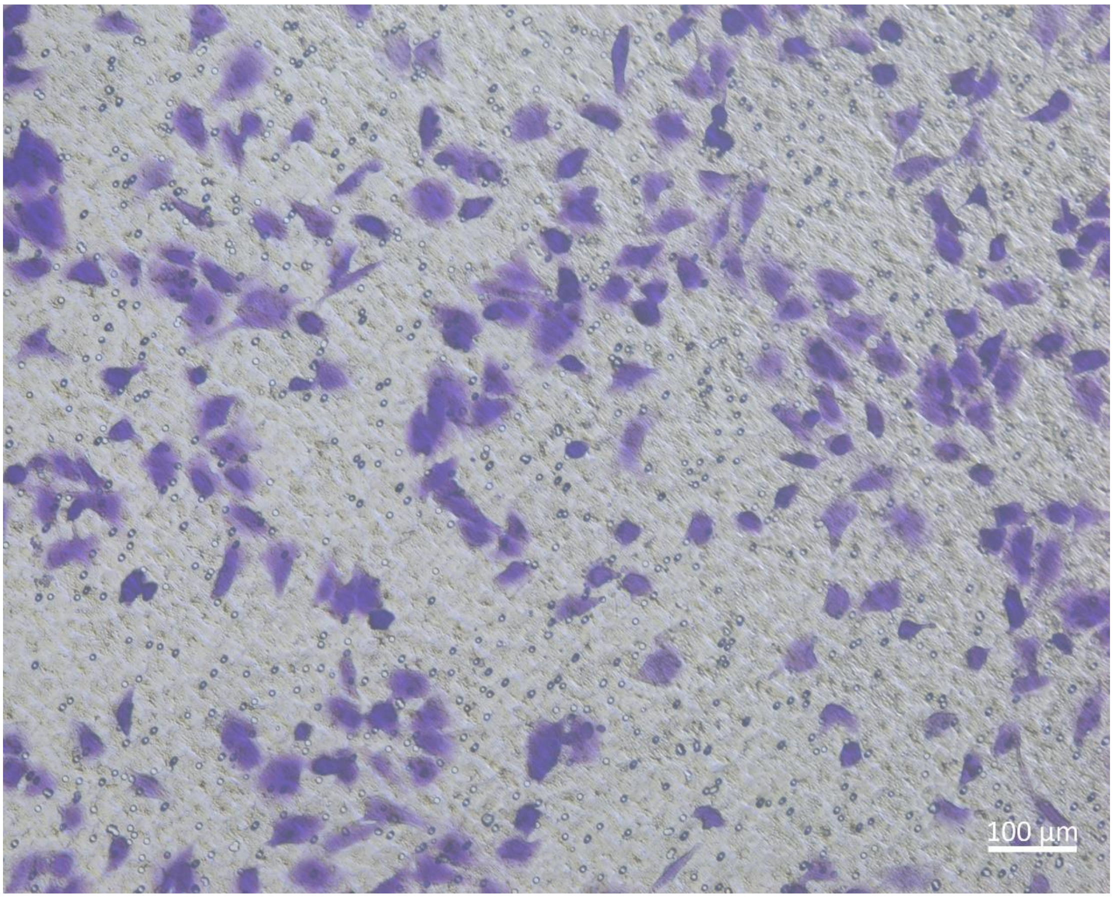

F3D-TPC1

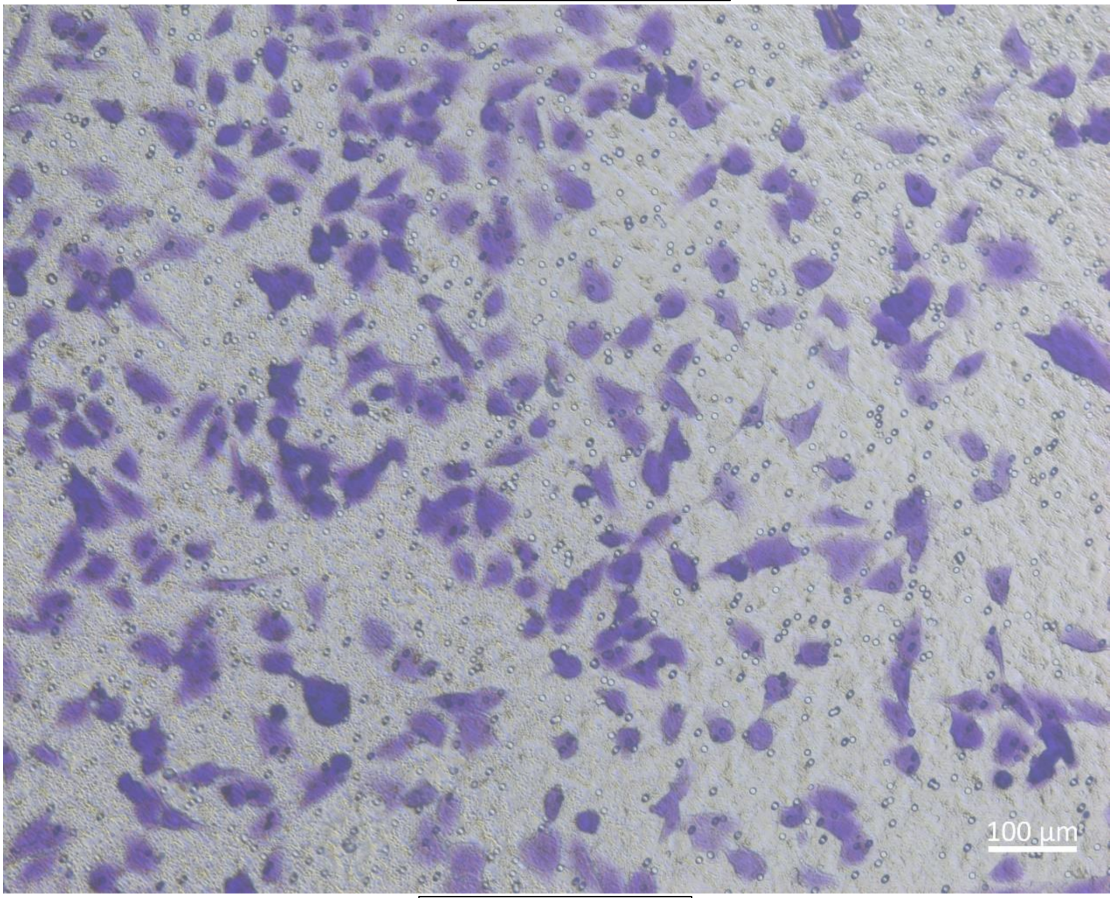

F3D-TPC1$^{Met-/-}$

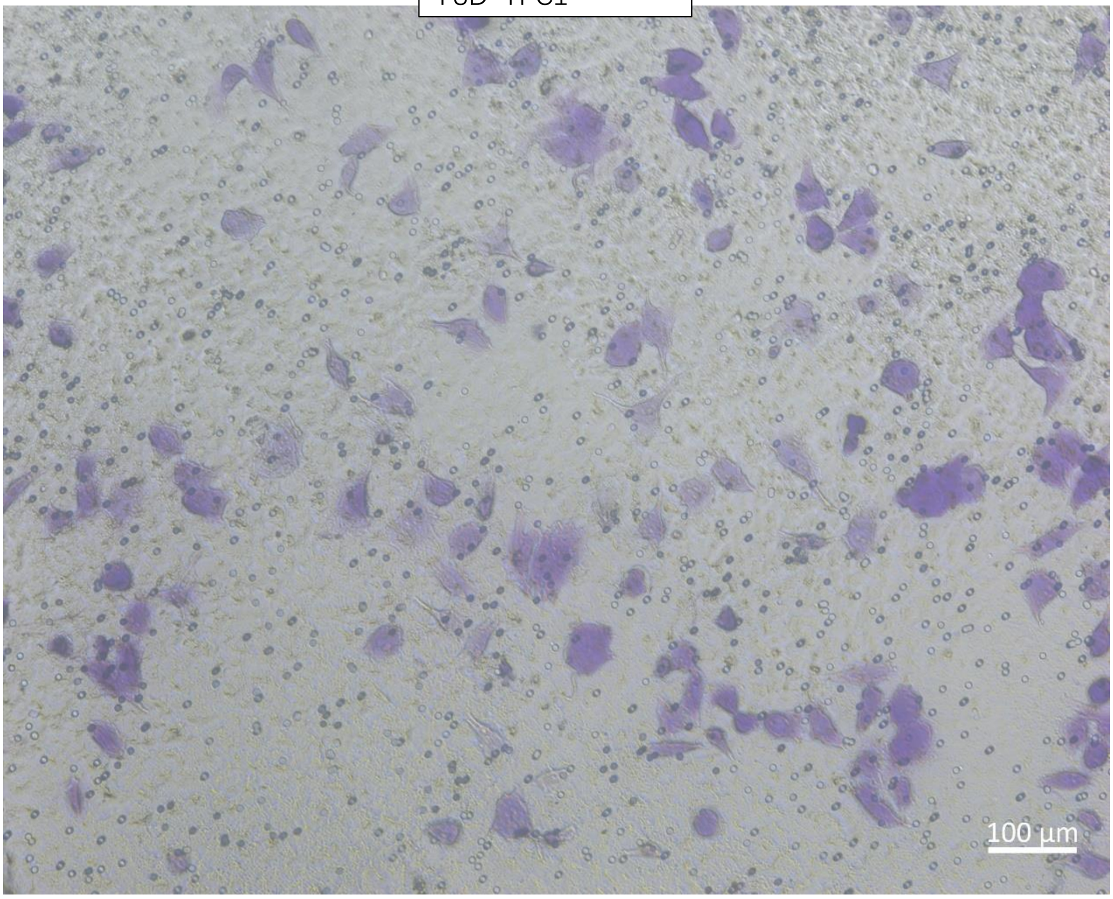

Supplement: Supplementary file 1 — Supplementary Material 1 [file 41598_2025_23587_MOESM1_ESM.pdf]
