# Supplementary figures and images for: Overexpressed MET drives aggressive thyroid cancer phenotypes and serves as a precision therapeutic target
Source: Sci Rep. 2025 Nov 13;15:39809. doi: 10.1038/s41598-025-23587-7 (PMC12615610; doi:10.1038/s41598-025-23587-7)

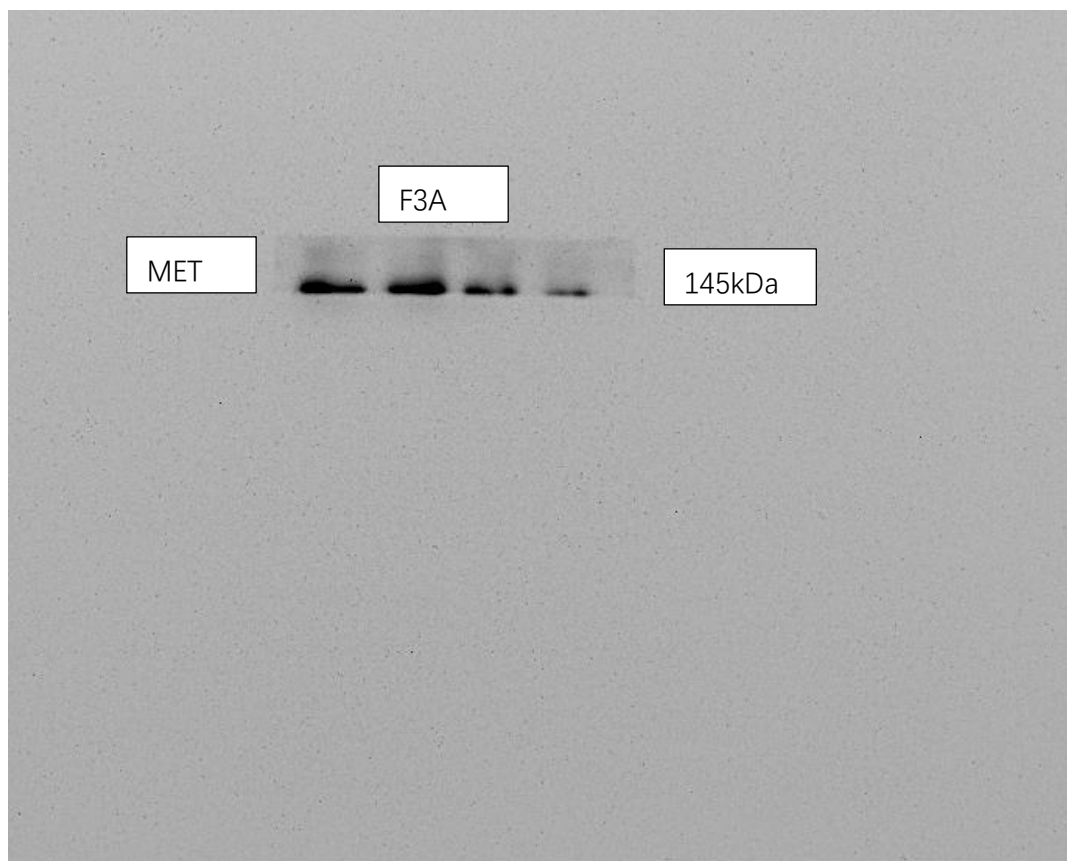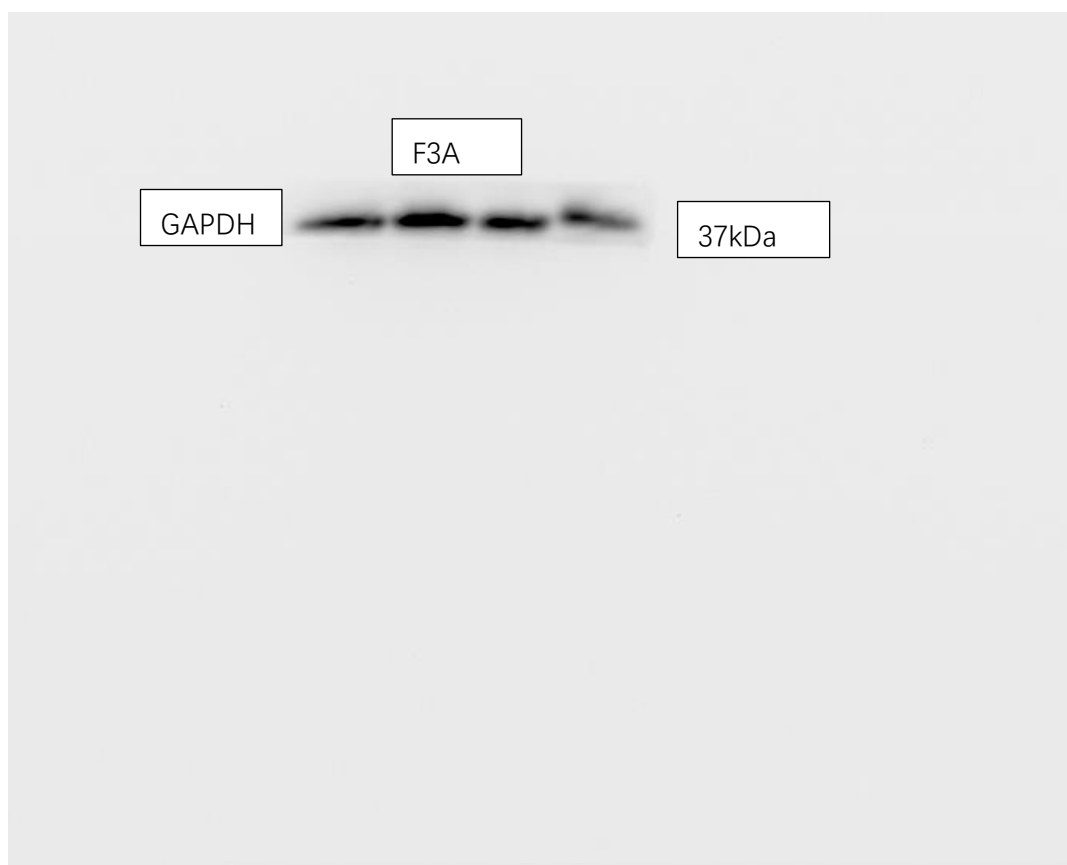

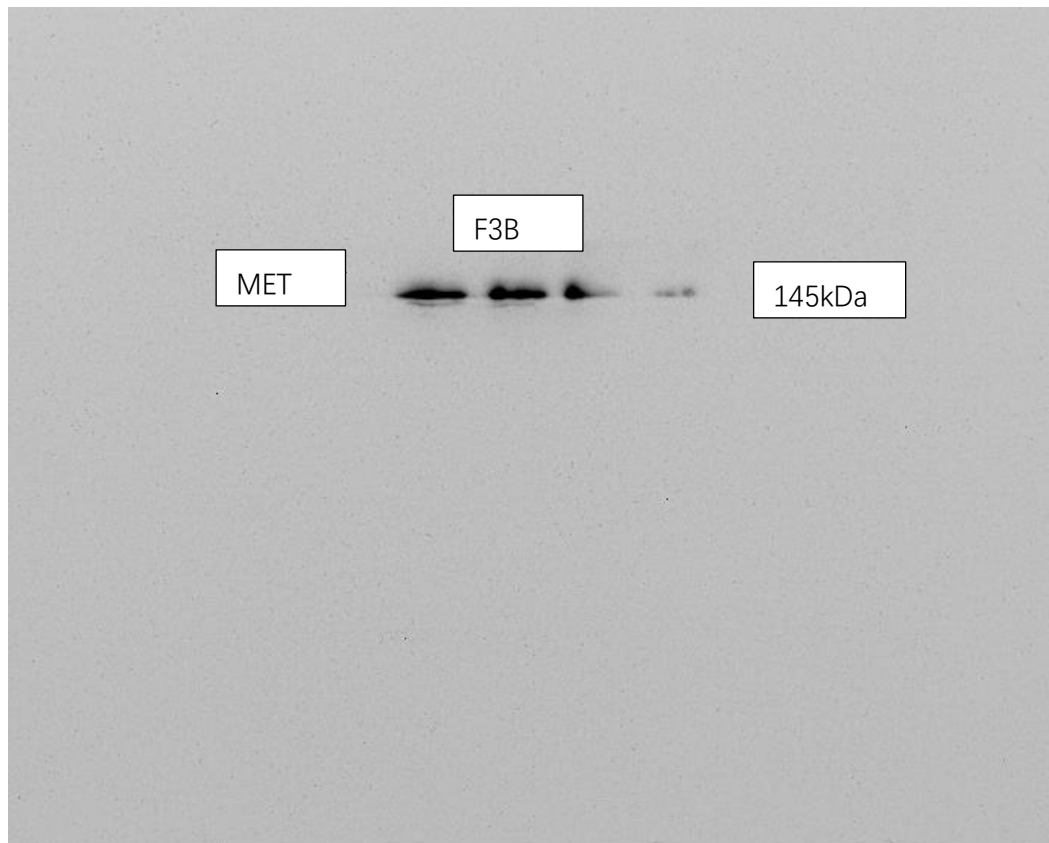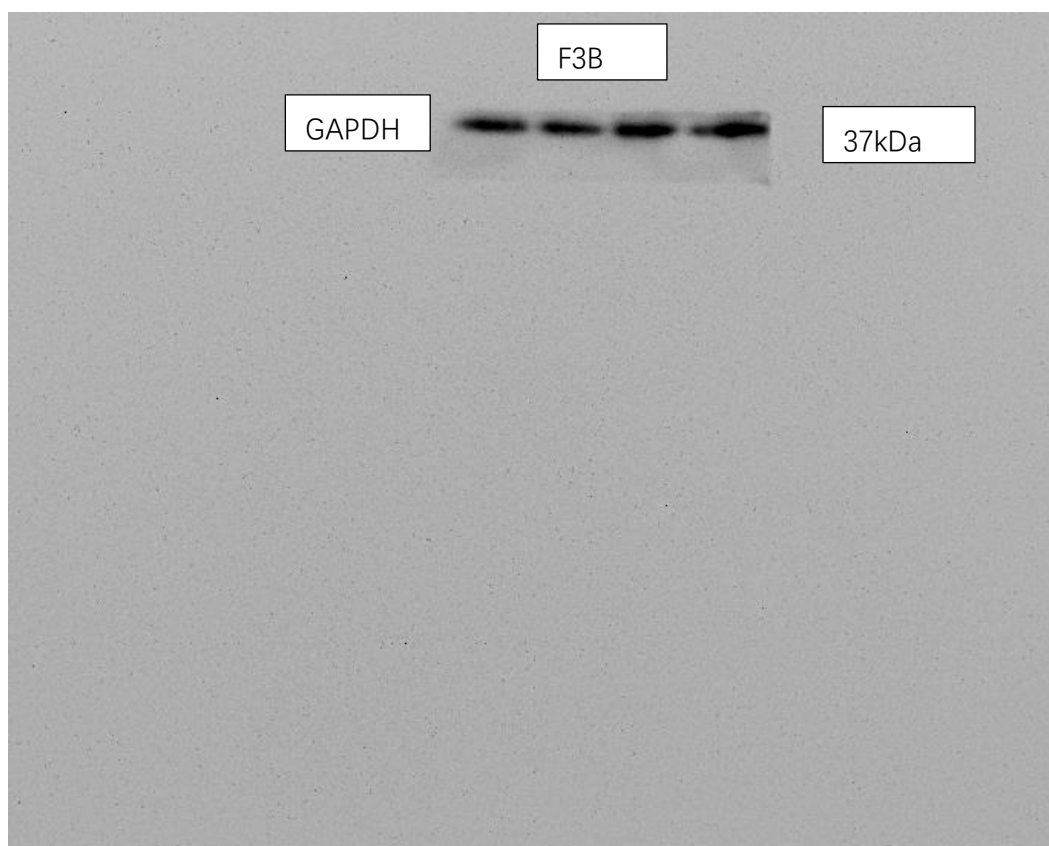

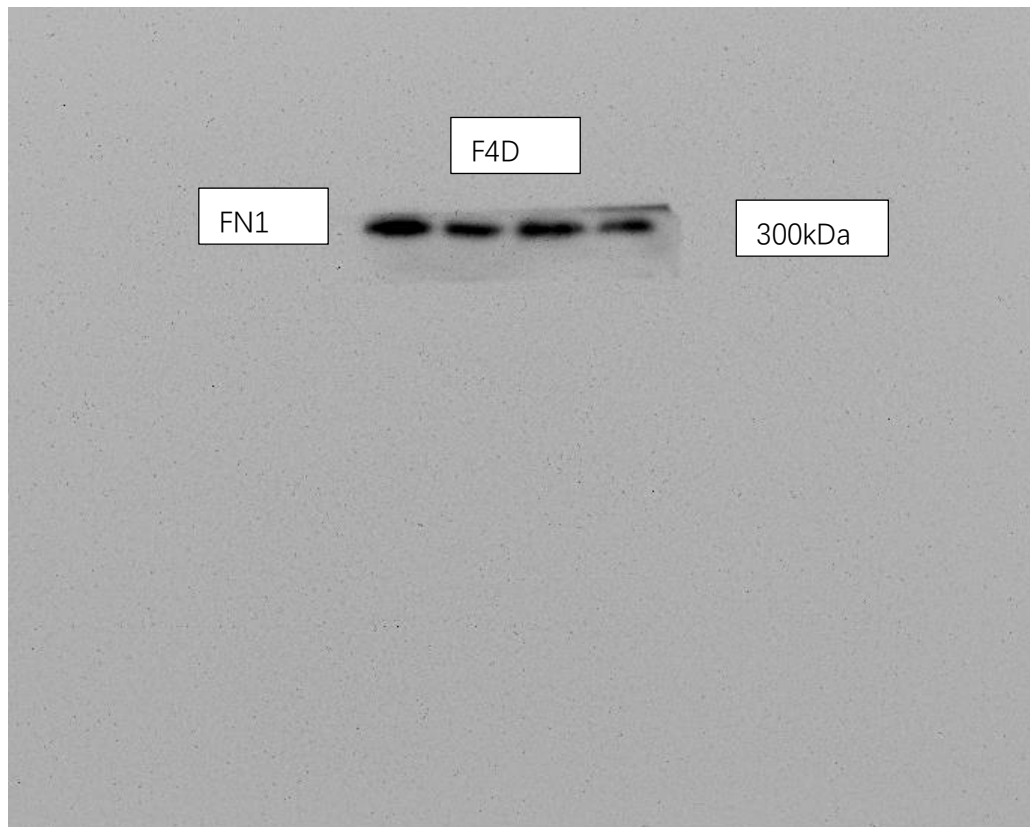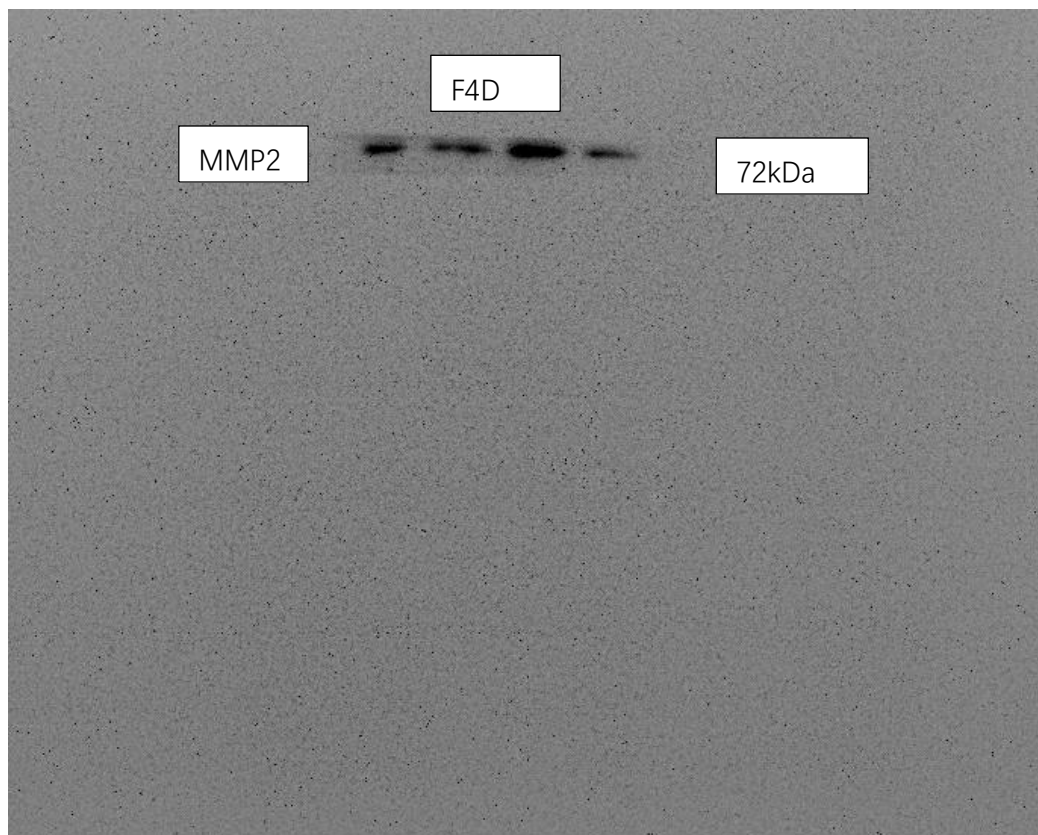

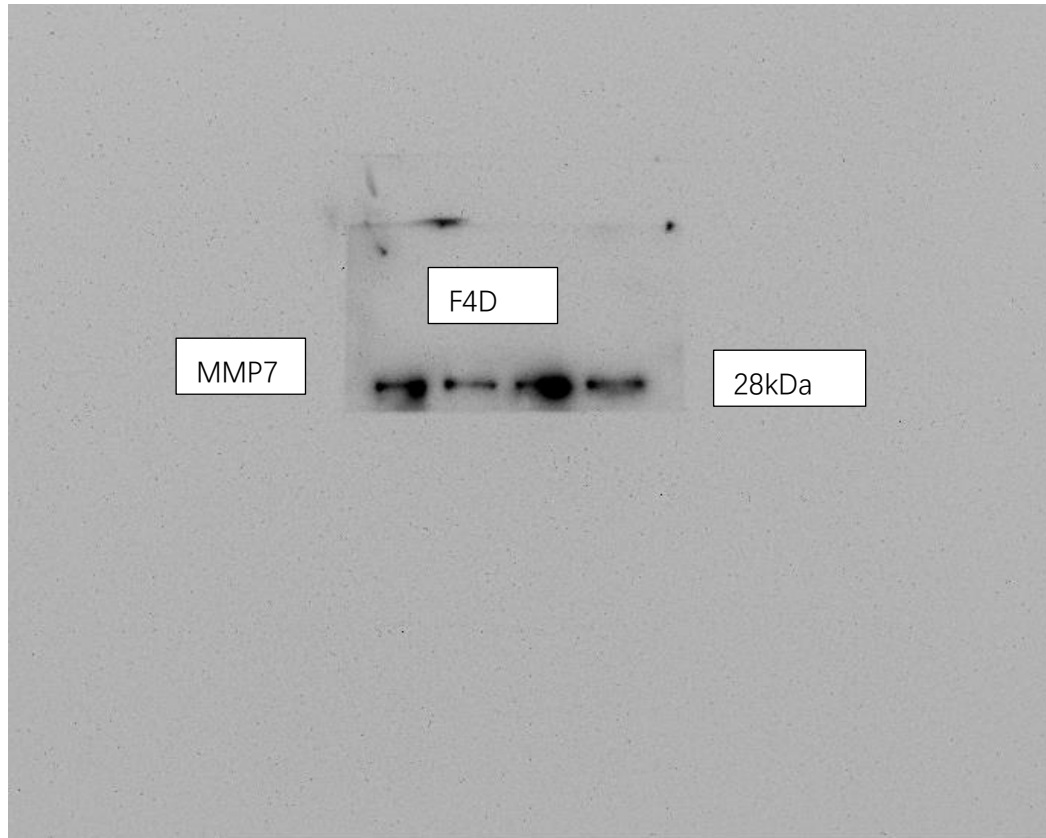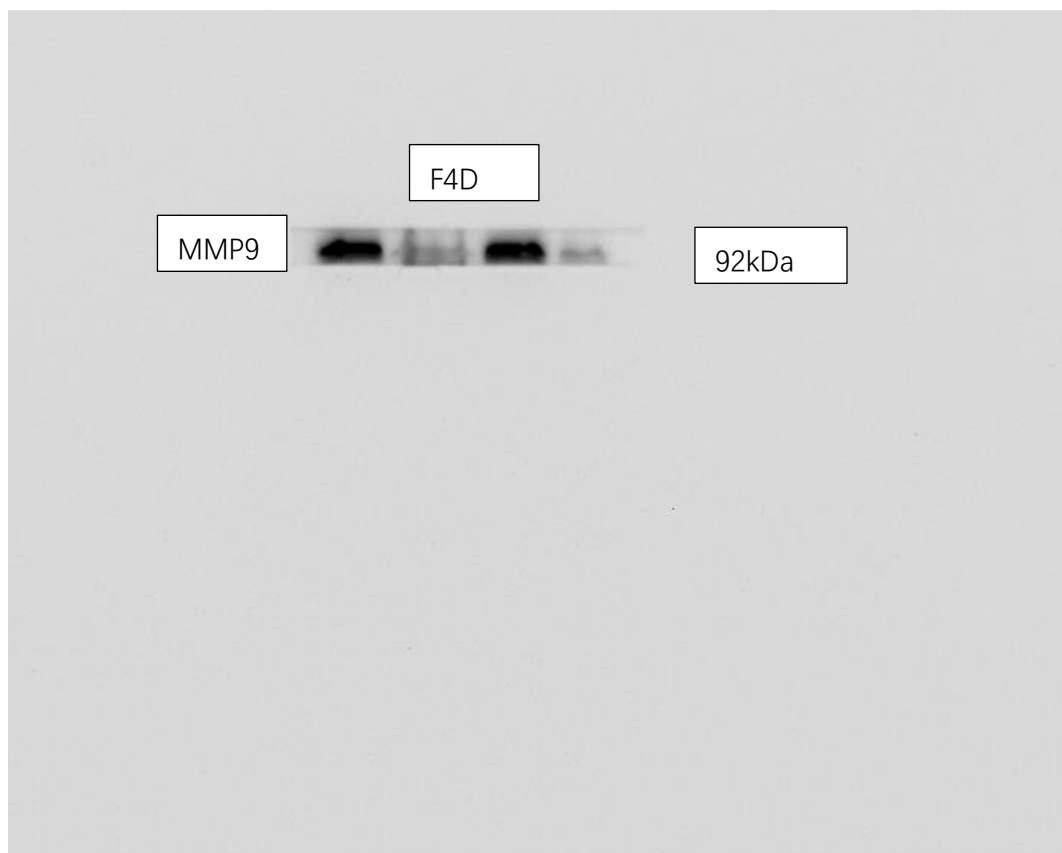

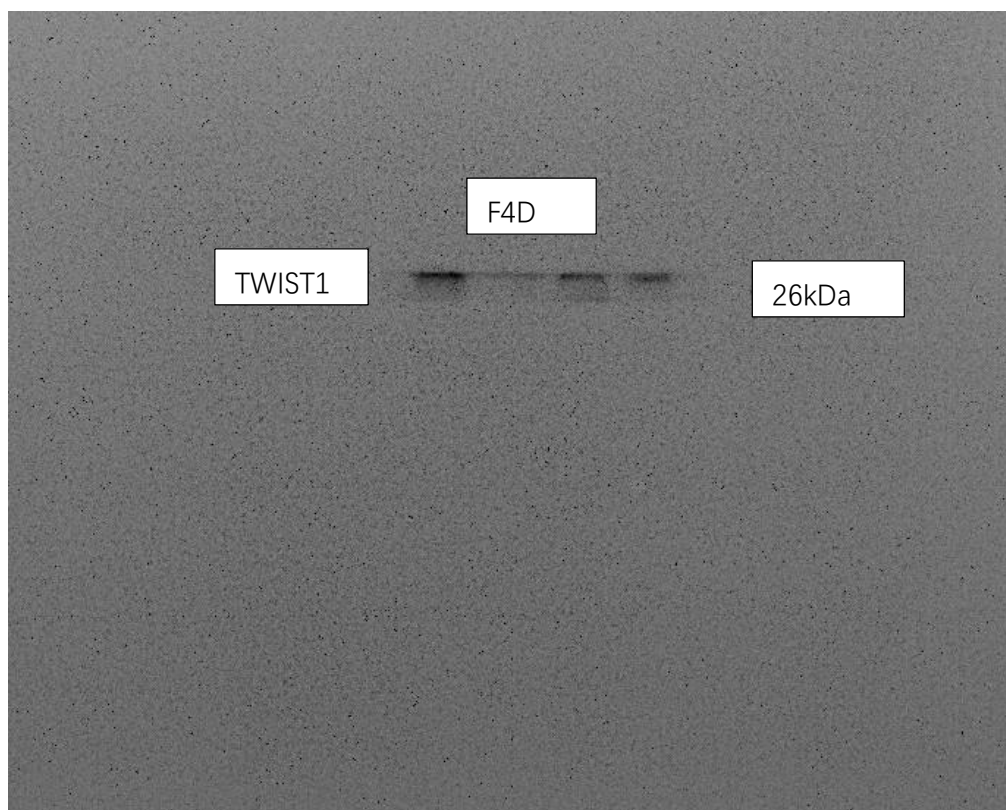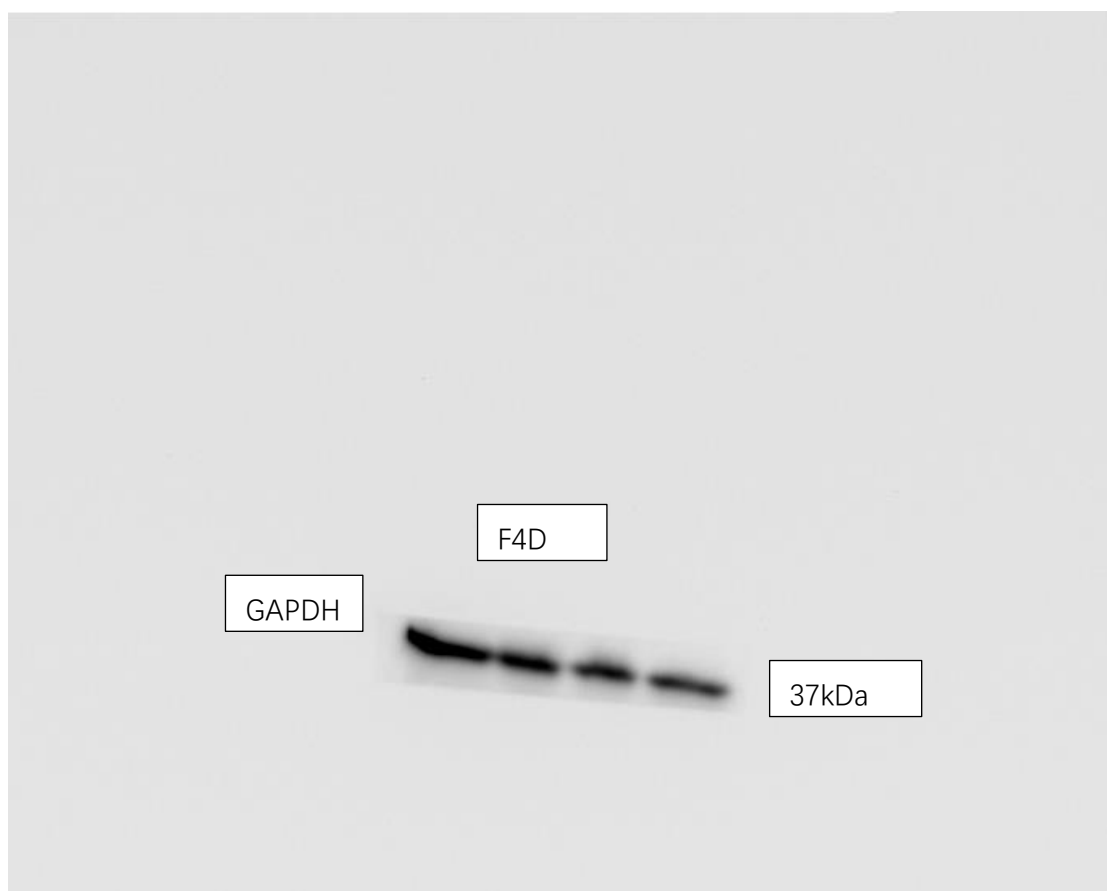

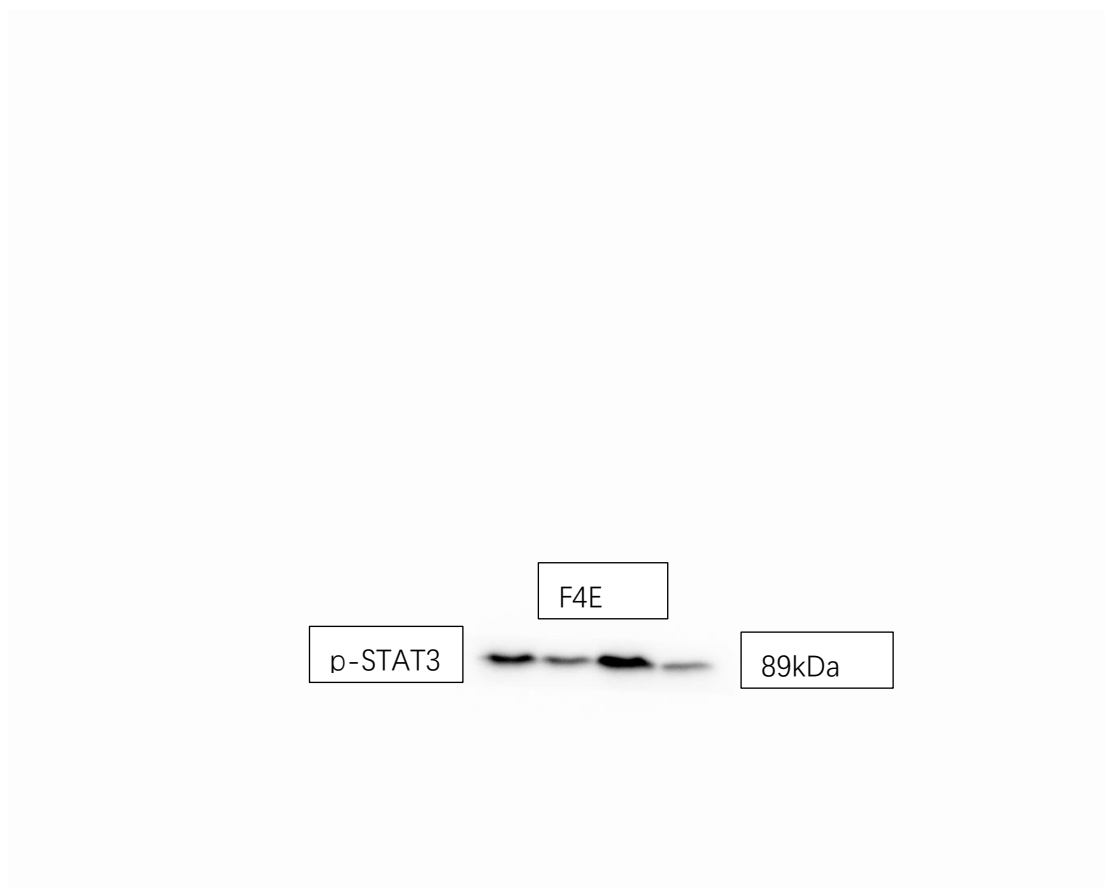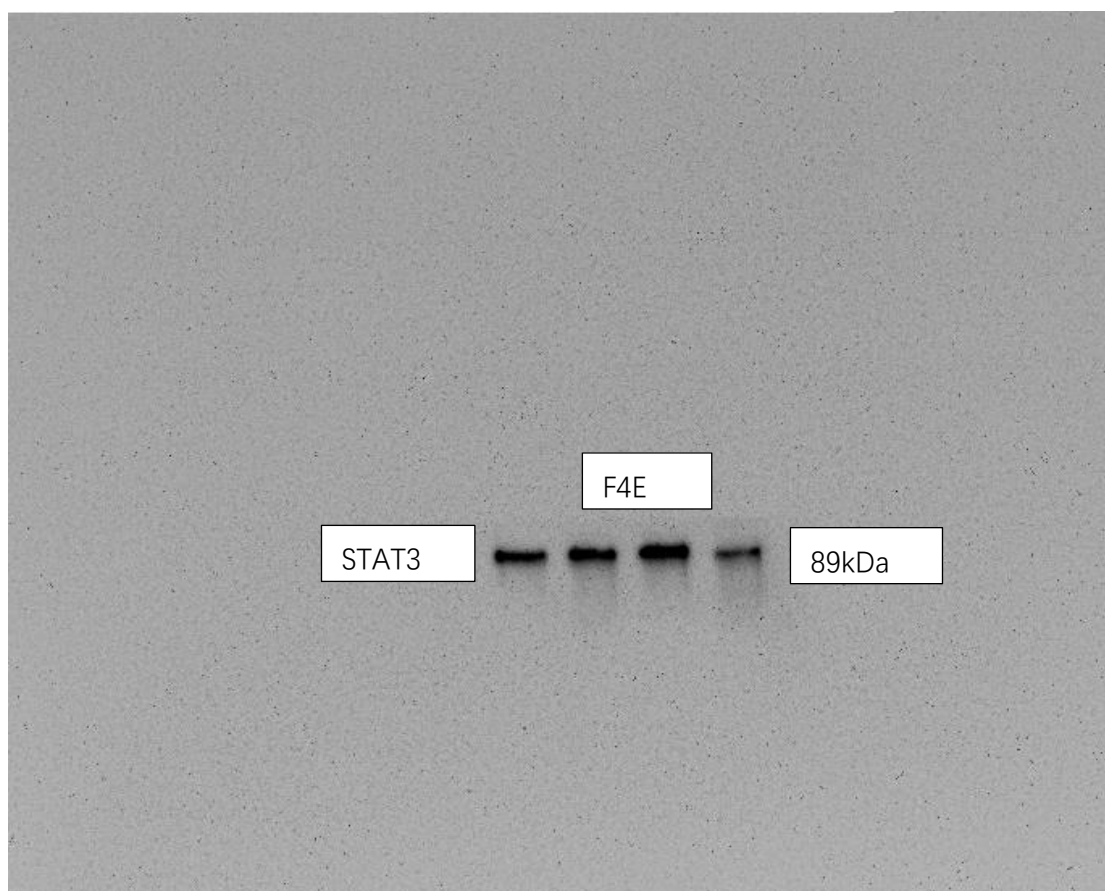

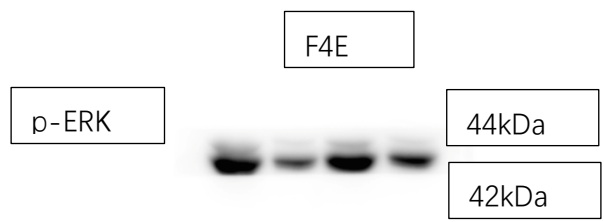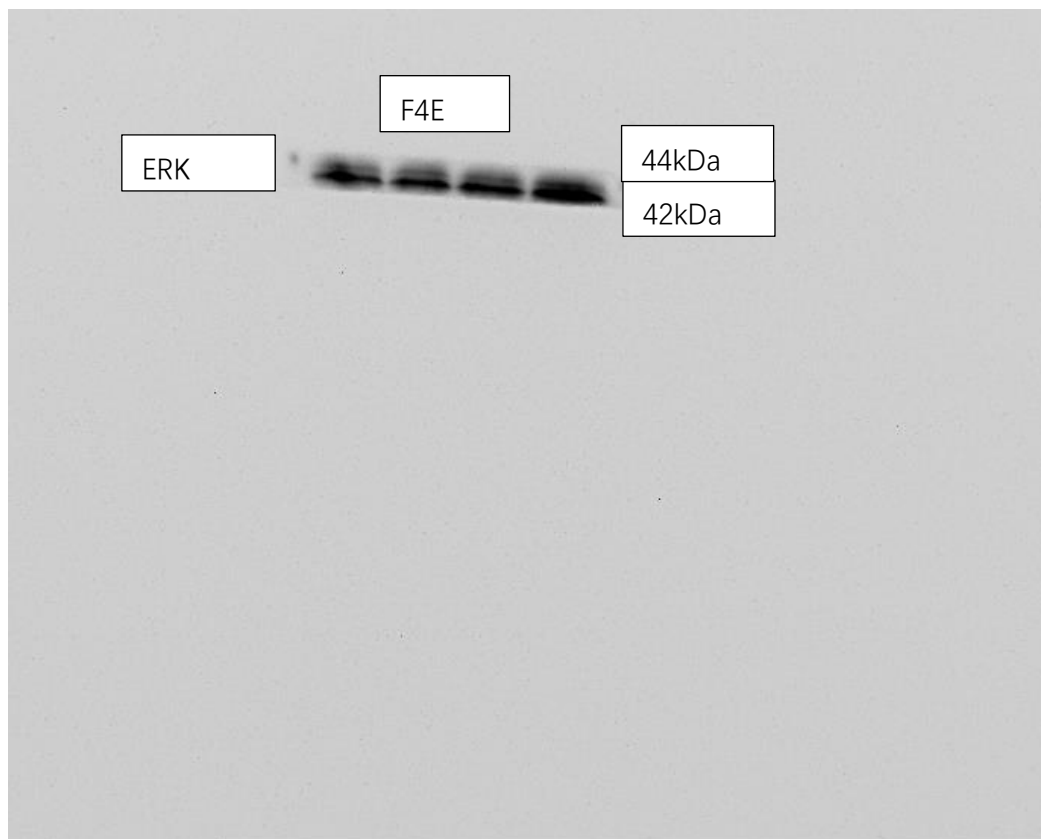

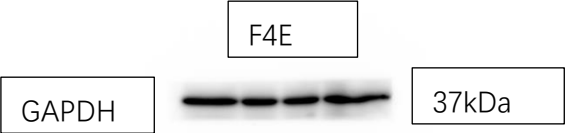

Supplement: Supplementary file 2 — Supplementary Material 2 [file 41598_2025_23587_MOESM2_ESM.pdf]
